# Supplementary material for: Biosynthesis of Sesquiterpenes in Basidiomycetes: A Review
Source: J Fungi (Basel). 2022 Aug 28;8(9):913. doi: 10.3390/jof8090913 (PMC9501842; doi:10.3390/jof8090913)
Supplement: Supplementary file 1 [file jof-08-00913-s001.zip › File S1.pdf]

>STC4 （Genbank ID:KAH0582448）
MVQFRIPDLLSCLPACIKATNADNDILQAGLVVIDQCHLTDHYKKDLKRAQIPHLAIR
AFPGSDLKYLRICVEYLIAAFLLDRLTDKPATAAQAQEWADIYKQYFRKTLQGTKGPAR
INQYLTKKCDIYPQAFSKTFEKTKGPAEIKYLTSHMSNTIKDPYWSCLVENNILLADGM
AKEAVDRENPGTEMDLETYIKVRRDTIGARQLFDLGRWIHELNITPETLTHPDIVRMEE
QFIDLISLANDLYSYKKEYLAKDAKHNYLTIALRDPTADLHENDLQGAINYTYDKFCQ
VLTDLEHQKKVLPRFGKSEEAKVDKYFWLMMNVVIGTIQWSLECERYGHFVDVDGP
NQGDVVFNL
>STC9 （Genbank ID:KAH0583476）
MFRFDHPSSFILQNICDITGAVFELKENPLREQANTAVLKWFKGFNVYDKAQGEKFIN
AGRFDIFAALSFPEADIEHLTTCLAFFLWAFSTDDLSDEGEYQSKPEKVRRGHEISCSILH
DDSAPQPSYPYAGMLWDLLRRLRANGKSGMYKRFKQAFLDWSSSQVQQSLNRNLDRI
PPVDEFILMRRCTIGAALVEAMVEYSLDLDIPSFVWEHPVIIGMSQVTSDIMTWPNDLC
SFNKEQADGDNQNFVCVLQHAHNLNLQEAVDLLTKMIADRVQDYVKLKKRLPSFGP
DIDPAVHKYVDALEQFVQGCVVWYYSSPRYFPDIDPRGKSKAEIHLLSKPISSDPVQMH
>STC15 （Genbank ID:KAG5341349）
MSAATSQLLPSALATKIILPDLVAHCDFTLRYNRHRKQITRETKRWLFKGGNLNGKKR
DAFHGLKAGLLTAMTYPDAAYPQLRVCNDFLTYLFHLDNLSDDMDNRGTRSTADVV
LNSLYHPHTYYGPERVGKMTRDYYKRMIVTASPGAQQRFIETFDFFFQSVTQQAIDRA
NGVIPDLESYIALRRDTSGCKPCWALIEYANNLNIPDEVMEHPIIVSLGEAANDLVTWS
NDIFSYNVEQSKGDTHNMIPVVMNEEGLDLQSAIDFVGNMCRQSIDRFVEDRTNLPS
WGPEIDKDVAVYVNGLADWIVGSLHWSFESERYFGKTGREVKANRVVNLLPRRA
>Cop1 （Genbank ID:XP_001832573）
MVNLSWYWQGQGNISKSIGPPSQTYTKSVLREQSMTFRMLALQSGLKSAASDHVSTSG
SGILRFLSRILPANTTRRCSACCTEMSSLDATIHPVLNFEDKKIVLPDLVSHCNFKLRVSR
HRKRITGETKRWLFKGDNLVGPARNKYHGLKAGLLTAMTYPDAAYPQLRLCNDFLTY
LFHIDNLSDDMDNRGTWSTANEVLNSLYHPYTYHGQARVGRMTRDYWRRMILTASP
GSQQRFIETFDFFFQSVTQQAIDRLTGEIPDLESYIALRRDTSGCKPCWALIEYANNLDLP
DEVMDHPVVRSLGEAANDLVTWSNDIFSFNVEQSKGDTHNMIPVVMHQEGLDLQSA
VDFVGEMLDHTSTCCGLESPLWLTPPLRIRRNAPRLYRVFL
>Cop2 （Genbank ID:XP_001836556）
MPSPAGALPKSFILPDLVNDCPFPLRVNPLCDEVGRLSEQWFLRHANYSPPRAVAFMA
LKAGELTAACYPDADAFHLRVSDDFMNFLFNADDWLDDFDIEDTYGLANCTVRALR
DPVNFITDKRAGLMTKSYFSRFLKTAGPRCTERFIQTLALYFESVVTQKQARNNGTLPD
LESYITIRRNNSGCKPCYALIEFCAGIDLPDEVINHPIIQSLEDASNDLIAWSNDIFSFNRE
QSRHDSFNMVSIVMHQKGFALQEAVNFVGELCKKAMERFQADKRNLPSWGPEIDGE
VAMYVDGLQNWIVGSLNWSIDGTERYFGKDGPGIKKHRKVKLFPKRPLKTPAVRVLA
>Cop3 （Genbank ID:XP_001832925）
MSTPSSSLTTDESPASFILPDLVSHCPFPLRYHPKGDEVAKQTVHWLDSNCPDLTAKERK
AMYGLQAGELTGYCYPYTTPERLRVVADFLNYLFHLDNISDGMMTRETAVLADVVM
NALWFPEDYRPTKGQAAEELNPGKLARDFWSRCIPDCGPGTQARFKETFGSFFEAVNI
QARARDEGVIPDLESYIDVRRDTSGCKPCWVLIEYALGIDLPDFVVEHPVIAALNQGTN
DLVTWSNDIFSYNVEQSKGDTHNMIIILMEHHGHTLQSAVDYVGSLCQQTINTFCENK
QQLPSWGPEIDDMVAKYVQGLEDWIVGSLHWSFQTRRYFGDEGQEIKQHRLVKLLTV

APPPPPPPPTPPPQSSDADTKKQKVKAQDGKGPVSDEEVWALVRAEQSKGSILESLFGF
LTTSLSRIFFGYF
FAYSH
>Cop4（Genbank ID:XP_001836356）
MRPTARQFTLPDLFSICPLQDATNPWYKQAAAESRAWINSYNIFTDRKRAFFIQGSNEL
LCSHVYAYAGYEQFRTCCDFVNLLFVVDEISDDQNGQDARATGRIFVNAMRDAHWD
DGSILAKITHEFRERFVRLAGPKTVRRFADLCESYTDCVAREAELRERNQVLGLNDFIA
LRRQNSAVLLCYSLVEYILGIDLDDEVYEDPTFAKAYWAACDFVCWANDVYSYDMEQ
AKGHTGNNVVTVLMKEKDLSLQEASDYIGRECEKQMRDYLEAKSQLLQSTDLPQEAV
RYIEALGYWMVGNLVWSFESQRYFGAQHERVKATHVVHLRPSSVLEASCDSDSDSDC
>Cop5（Genbank ID:XP_001834007）
MVGSYTGKVIHVPALLESWPWPAAINPLYEQVQEESTSWFRKFDLYRDRKKQAIHDHL
DTAKFGASVCPKADYALLRLATDYLHLGFWIDYFFDTSPSDVIRQLTESIAHLLESGDPR
LDSSSPQSHIACMEILRDFRKRIETFNPSQEDLRRFVKEYRGFLEAELTQAIDHENKVIRD
IESYLSIRRSTIAIRGIALLGLALGIPQEILDDPYTDTLTNACLDMVIIQNDAYSWNVEQV
RKADGHNIITVLMKQRDIDVQEAYEHAAQLHRETQEHFLELHAKRPDWGNEGSIQAF
FDGLGEFVRGVDEWSSMCLGEHALSVGAGFLK
>Cop6（Genbank ID:XP_001832549）
MPAALPYNVSRDNKWDIKKIIQDFFKRCDVPYQVIPYDTELWNACLKRAKEKGYPVE
PDSPMSLYRSFKVGVVITRTSYGHIQDYEILIWVATFTAFVTYADDAFQEDIQHLHSFAR
TFLQNEKHEHPVLEAFAQFLRESSIRFSHFVANTVVSSALRFMMSIALEFEGQNVSVSTE
AREYPGYIRILSGLSDIYALFAFPMDLPRSTYIQAFPEQIDYINGTNDLLSFYKEELDCETV
NFISAAATSQQVSKLEVLRNAAEKAAYSYDVVVNVLKPYPEALAAWKSFARGFCYFHT
SSPRYRLGEMFHDFEHDLVCKCASCTEI
>AcTPS4（JGI ID:M_Fcontig40411）
MSAQQFTLPDLLAVCPLKDATNPHYAQAAAESTAWVKSYNIFDARKLAFLLQGSSELL
VSHAYPYAPYEQFRTCCDFVNLLFVVDEVSDDQNGKDARRTGEVYLNVMRYPDWDD
GSALAKMTREFKQRLLAFAGPNSYRRFLMHCDDYVNAVAREAEYRERGEVLDIDAFQ
TLRRENSAIRLCFGLFEFALGIDLPDFVFQDPHFMTLYWSAADMVCWSNDVYSYNME
QAKGHTGNNIVTVLMRQKSIGLQEAADLVGAHFSALMGRFVETKKQLPSFGAAALD
DAVAKYVAAMEHWVIGNLEWSFESQRYFGAEHTRVKATRVVVLSPADEN
>AcTPS5（JGI ID:M_Fcontig40579）
MAVTPAPVNGSDSTKEIILKFPDFISPIPYPLRCHPQEREVSRQSEEWLLSMANFSEKQR
AKFLTLNAGLLSGWCYIDCTFDELRVCTDFMNFLFTLDDWTDEFDTTGTRGLAECVM
NTLYFPDTYKSDTAAHRLTKSFWERMRATAGPGCQQRLLSTLDTYFQAIMQQAADRG
SRNIPDLEEYILLRRDTSGCKTGFAFIEYAANIDLPDEVIEHPIIKAMSDSTNDLVSWAND
VLSYNAEQSRGDTHNLVCVLMHQNNVDRQEAIEQAGELWRKTLDYYFECHKALPSW
GPEIDRAVALYVQGLDDWIIANAEWSFETERYFGKEGPTVKKTRQIPLL
>AcTPS9（JGI ID:M_Fcontig47706）
MSSPSSFVLPDLHAVTPFKGSFNPHYPEAAAESSEWVNSYKVLSDKKRAFFLQGGSELL
CAHAYPYAGYQQFRTTCDFVNLLFTVDEISDDQNGKGAYETGLTFYNAMSNPAYDDG
TVLCKMTKEFTARLLEHCGPQTYRRFIKHCKDYIEAVAVEADLRERGEVLDLEAYQTLR
RENSAVRFCFGLAGYALGIDLPDEVVEHPAFMAMHLSTVDMVCWSNDLYSYNMEQA
MGHTGNNVITVLMQHKGLDLQGAADYTGVHFKGLIDTFLDAKRSLPSWGPKLDGEV

AQYAMAMETWVIGNLNWSFETQRYFGHARHEIKRTRVVQLYPRRIVEESSDEE
DN
>CpSTS1（Genbank ID:LC436345.1）
MPAAIPKFYTLPDTLRNWPWKREISPYYRQCQAESVAWLESFRPFSPKAQVAFNKCDFS
LVSALCFPKGSPYNLRSVCDLMHTFFTLDEYTDYLDLEGVKTLCEATMDAIRNPDKPR
PEGEHFIGEVARQFWARARVNATPACEERFVKSWRTYLNSVIQQAERRDAKYICTMEE
YLHARRDNIGSDPSFALLEITLEVDLPHEVMEHPTIVALARDTTDMIVLANDMCSYKK
EIIADDANYNAVTVVMHNHNTNVDGGIQWISDYHDTIVDHFLRLREDVRLKQNGFPS
WGARIDREVEAYVEGLGLWIRGHDEWNFGSGRYFGDEGLEVQKSRIVECTVSADPFTP
FLIQQEEEVDENAA
>CpSTS2（Genbank ID:LC436346.1）
MMQFYLPALVEQCPIEGGTNPHYEQGAAESRAWINGYNVFTDRKRAFFILGSNELLCS
HVYYYAGSEEFRTSCDFVNVLFIFDELSDEQTGKDALETGQIFLNAMKDERWDDRSKF
SSMTKEFRKRFLRRSGPRGTARFLKHWETYCAAVIREAELREMDEVLDLEDFINLRREN
SAVRLCYGLIEYCYGIDLPEAVYEDSTFMDIYWAAVDLVCWTNDVYSYDMEQSKGIAG
NNIVTVLMRNRNMSLQQASNYIGQHCETLMDRFVSSQVRLPSWGPVVDREVRLYIQG
LGAWIKGNLDWSFETQRYFGPMHEEVKSTRLVTLRPRERIEECDSDSDSDFE
>CpSTS3（Genbank ID:LC436347.1）
MVATTASTQPDHFVLPDLVSHCSFPLVYHTDGDRIAAQSVNWLDSNCPDLNAKQRVA
LRGLQAGELTAFCYNTCTPERLRVVSDFMNYLFHLDNISDGMMTRETDVLADVVMN
ALWFSGKYMPTKEQSADELNPGKLARDFWARCIPDAGPGCQARFKETLELFFEAVNIQ
ARARDDDVVPDLESYIDVRRDTSGCKPCWALIEYALDIDLPDFVVEHPIIEALNQSTND
LVTWSNDIFSYNVEQSRGDTHNMIVILMKYHGHTLQSAVDYVGDLCQKTIDDFQANR
QKLPSWGAEVDEMVQRYVVGLQDWIVGSLHWSFQTHRYFGADGANVKKNRIVKLL
PLKA
>CpSTS4（Genbank ID:LC436348.1）
MSESNQHLRIPHTLTAWPWPRTLNPYYQTVKAESSAWLESFKAFDPKAQAGFNSCDF
NLLASLAYPLASKEHLRTGCDLMNLFFVIDEYTDIEDEVHAAMVASVTMDALRNPFKP
RPRGEIIIGEIARQFWARTVPTITEASHRRFIETFDTYLQSVVVQARDRSKQHIRSIQDYL
HMRRDNIGAKPSFAILELSLDLPDYVMSHPSIQTATVTAIDMLIIGNDLCSFRNEHARG
DDTHNILTIARHEFGQGLGHAVNWIESYNKSLRRSFLSAIERVPSWGEEIDTQVAEYLY
GLANWVRANDCWSFESHRYFGKHGREIQVHRVVDLACGHPECVQDTDNEVEKPAT
KGLLYEVEKKTYVGPLQVNTKDPIAFRSDFPLSARPKDITSTPTRLTCVALLCTLSTFIGW
LVKL
>CpSTS5（Genbank ID:LC436349.1）
MVATTASTQPDHFVLPDLVSHCSFPLVYHTDGDRIAAQSVNWLDSNCPDLNAKQRVA
LRGLQAGELTAFCYNTCTPERLRVVSDFMNYLFHLDNISDGMMTRETDVLADVVMN
ALWFSGKYMPTKEQSADELNPGKLARDFWARCIPDAGPGCQARFKETLELFFEAVNIQ
ARARDDDVVPDLESYIDVRRDTSGCKPCWALIEYALDIDLPDFVVEHPIIEALNQSTND
LVTWSNDIFSYNVEQSRGDTHNMIVILMKYHGHTLQSAVDYVGDLCQKTIDDFQANR
QKLPSWGAEVDEMVQRYVVGLQDWIVGSLHWSFQTHRYFGADGANVKKNRIVKLL
PLKA
>CpSTS6（Genbank ID:LC436350.1）
MLTTPYFTIPDTLRNWPWKRVLNPHQKVCEEEAADWMRSCGAFTPKSQNAFDRCSF

GLLGSLAYPRLGRDGLRIACDLMNMFFVIDEYSDVASGREARLQADIVMDALYNPLVP
RPVGEWIGGEVTRQFWANAIKTATPSSQRRFVRNFQRYVDAVVQQAQDREAHCIRDV
KSYFILRRQTIGAIPSLDLLTLEMDLEDEVLDHPIIAKLLELCVDMILIGNDLYSYNVEQA
RGDDTHNFVRIVKDERKCNLNDALRWISDYHDRLADEFLNLMHNLPSFGSKLIDEQV
KTYVDGLGNWVRANECWSFESERYFGKKGKLYQKSRRVRLLPSSIALQGQNIDPQAA
VEISDLPAAAGIIV
>CpSTS7（Genbank ID:LC436351.1）
MTTSGFRIPNLVVAWPWLRAVNPHMDQTSQNSSSWLENFHAYPPATQENFNRCDFGL
LAALTYPIASKEHLRTGTDFMNLLFFIDEITEIGDETFSTEVLYIAMDALRNPSKLRPKGE
HVVGEITRQFWERSIPHITATSHRRFVCALEAYLKSNVVQARDRSSQHIRRIEEYFSTRR
DNIGTKPAFVLLELGLDIPDCVMTHPSIQSAVTSLTDMLIIANDVFSFKVEHSRDDDAN
NVLKVVMCELRCNLARAIEWADERNQQLRTTFLSSIAEVPSWGGQVDIQVSEYLYGLA
NWVRANECWSFESQRYFGTHGPTVQKDRWVSLDCGHPQCITGGTTTPRTSYRPYLVG
MLGIATYALSQYGLMSRATLLRR
>CpSTS8（Genbank ID:LC436352.1）
MSNTFIVLPNLEETVYSAFPDHGLNPHYDAVCPHSRAWIKSYSDPIFGPKMRDFMEKC
DFELFAAYICPRASPEALRTSMDIGNLLWLYDEYTDAEDSVTVKKAEAIVVRTLQDRSF
DDGSWICRLVKEFLDLHVRKKAGPNVSRRFVGHFVDYVVRVSDEATQRERHEVLDIEA
YVERRRESGAIRLTFDLIESGLHIDLPQYVHEDPAFIAGYNATMDLACWVNDVYSYNM
EQAKGHEASNILTVLMKYEHLDLQAAVEYVAKHCEVLAAQFIEAHANLLARSDPNFS
EDAARVLDALGDAVIGNDRWSFETERYFGKDYKAVKQSRIVKLAGRAEGKHALRN
>CpSTS9（Genbank ID:LC436353.1）
MTVLQFVFSVFTSSKPQTFFLPRLDETFKVLPNNGLNPHFSTVRPQSRAWIKQYDGEVC
GPKMRAFMDNCNFELSNAYTYPYAEPAGLRAAMDLTNILWLYDEYTDTEDGASAERA
AVIVNRALKEPGFNDGSWICRMMQDFRRRHIDKAGPDVARRFITNFCNYVDRVAREA
ILREKNEVLDIPSYILFRRETSAVKTCFDIVEYCLGLNLPQYVHDDPVFVSGYNAAMDLV
FWANDLFSYNMEQAKGHSGANIVTVIMKSKRVDLQTAADFVAGYCEALTAQLLEAK
QILSSRSDPAYSKDAVRVLEAFGDWVRGNDDWSFATERYFGKENNAVRESRIVVVRAP
FDETVKLVE
>CpSTS10（Genbank ID:LC436354.1）
MAPSYQLPDLPSLSRSFELRANPACKITTKASEASLIESKSTNGSHVLSSLERERLAAMK
VGLLAAICFPGSDPTQLRLLTDFLTLTVLATRRVKYATLSPQVLTYWTTVDDNDGVWSL
VHHDMFNCLKEPLERLASKANQNWKARFGSSVKSFRIAQCSSVTEDMDILDPEIETYL
SMNRDLSGLSMIFDLFELTQNLTLTVTDETITRTLDKLKVLATDIVSCSVDVAAFNYDQ
ARGNEKNLISLLMRHKRLSVQGALNYAGTLIKQYIDAFMAEERSLLDPTPSPPSNGSSLI
PSWIPFTPLVASATAIPPSTPPDPVSKADLTVYVQMLRDCIVGTLNWIYETDLYFGKKGE
EIRTFGWIFLSPKVQNNDSEREVPGDNVLP
>CpSTS11（Genbank ID:LC436355.1）
MSQKSPSTFRIPDLEAIFSAFPDEGTSPYYDDVLPEARAWIKLYQDQVYGPKMTEFIDRC
KIELITYYVHPVASRSCVRAMMDLHNLFWLYDEATDVQSGQTAQETAKVVRNSLTSPE
FNDGSWLCEMLQDFRKRHLDGVRSSSFVARFIEHFCFYTDRVADEAIYREKQRVLDIPS
YMAFRRETAAVRVVMDTVEYCAELELPRTVLDDPVFQVAYDAALDLAFGTNDIHSYN
MEQSKDHSGANVITVIMKERGLDVQGAMDYFGGYCEALTAQFLDAKRKIEKREGQE
WKDAVLILDGYTHFLTGQVRWGFATERYFGKKNKEVQETRVVELRAPFVDHVDLAD

>CpSTS12（Genbank ID:LC436356.1）
MESLPVTLVLPRLDDIFDDLPNNVVNPNYSIACPASRLWIEQYGTQIYGPKMQAFMNN
CNFELSTTYTYPYADAKRLRATMDLVNILWLYDEYTDRASGAHAKEMASIVYQALSGQ
QVAANSWVGCMMQDFYRQHIEKAGPNTSRRFVDHFCRYAQQVGEEASLREQRQILN
MHEYIDFRRETSGVRSCFDLVEYCLGIDLPQFVHDDPIFTMGYNAAMDLVFWVNDLYS
YNMEQAKGHGAANVVTVIMKSKSLGLQAAVDHLADACEVLTAQFLEAKSRLSKHPE
SIFSKEAVKVLDAYGDWVRGNEEWSFVTERYFGKENKAVRQSRMVKIKTPFGEMAPFS
GRKNYL
>CpSTS13（Genbank ID:LC436357.1）
MPQDPPTTFRIPDLETIFSVFPDEGTNPHYDDVFPEAREWILRCKVELLTCYIYPSASKSR
LRSMMDLYNLFWIYDETTDVQTGQEAKETAQVVRNALTNPDFSDDSWLCAILQDFRR
RNLDGIMSPGFVLRFIDHFCDYANGVADEALFREKDLMLDIQEYLKFRREAAAVRVVL
DSVEYCLDLELEQNVMDDPVFQMAYNAALDLAFGTNDIQSYNMEQAKQHKGANIIS
VIMRARTLDLQGAMDYFGGYCQALTAQFLDAKCIIEKRVDRPEWKDAVRILEGYGCFL
AGQVRWGFTTERYFGKKNKEVESTKVVELRAPFVDHVHISD
>CpSTS14（Genbank ID:LC436358.1）
MNVMNTPITTATMPVSPPCPNPDPTFQTRLSARWHPSTAEIVPKVRQYIVQNWPWSSE
ESKRRYLTGNAEDGVMYAFPGVKNDRIEAVTTWLALHFLIDDYILESVSDSAASGLDP
GKKKEAIQRLFGIMRRLVRPNVNNPVEVMVDHVASSFLSCSSEDEVAQRHARQILESTI
QFITAAENGDGKATAMGDLNSYLAYRMVEAGVFLSLDLAFWGGEIYIPSHISNDPQIRS
FFKLVSDHLVLVNDIYSYKVEQDRSEGVGGAFNAVSVLMRSKHVDAQDAMGIIKTQLT
TLEEATLEEAAVLRDRYAEKGEEEEVVDKMIQTILEMMAGNCEWSKFCGRYNSSPVSD
EASVGTDRALEQGTTSLS
> CpSTS15（Genbank ID:LC436359.1）
MSSHRPTQQPNPLVDPPDFVSKFLAHDREDRDEIITSVQAYFLAHWDWPSPTRKAWYS
KADLEDWTTLMCPAGPSERAWAFTAYVTFWFLYDDLMEIMGPEELGSSIPRIARILHGE
EGLEDMTTPEFILKEICLKINTIAPGKRIFEATLVYMKAATAKQERLQSLTLGFDAYLKYR
MLDAACWLTLEGAYWVQEITIPEHLKTEKIWLLQELSLFHGILINDLFSYRKEVKASSQ
EVQDHDKTLYNGLVILMQSYQFTLLEALEEMKKKIWDYEDEFMAVLDDVRETHKSSA
EDREFIERLAVALMDVIGGNVAWSACCGRYNRL
>CpSTS16（Genbank ID:LC436360.1）
MPLPVTFRLPDLSSIFSIFPDLGINSHYECTYPESREWIAQYHTGVYGPKMRDFMERGKI
ELLGAYTYPYASKERLRFVMDLHNVSWLFDESSDVKTGQQAGVTAVVFRRALIDPEFN
DGSWLCHMLKDFRQRHLDNVMSHAFVEMFIRDFIGYADGMSAEACYRDLNKVLDIA
GYVKLRREAGAVRLALHSVEYCLEKELPSYVREDPAFVIAYNGALDLGYIVNDIHSYN
MEQSKGHGHHAANIVTVFMEAQNIGLQAAMDYAGGFCHGLVLQILEAKELLAARSD
PVFSNDAVKVIEGCINFFKGQDIWDFESERFFGKSKDAVRKTKVVNLRALFEDSVNLNE
>CpSTS17（Genbank ID:LC436361.1）
MNSDLQSTLASLDTTYTCRFRARYHAKSEQVMTVVQDYFVKRWPWRSEQAKQLYIKT
NLEEATCICFPTTLDDRIETVVTWFCYMHILDDIIEGLEANKATKLVDRLSNIILGTLDPD
PEDRLGVMAADVCSRFRLGENDGEEKHGLDIISESCKLLRHTSSADSKLQSLSTYDTFIE
WRELDVGLWFSTSLFLWGCGIYMRLHMSNDPEVRQLLRVSGRHIAVANDLFSYRVEAL
RAGQTSQILLNTIAIIAKEKQVDPQTAMNMTKQRLSEMEEEVEVLIGKLRDRYTGEEGE
LIERLFVVCKGMMAGNCEWSSICFRYNGAQRAVAA

>CpSTS18（Genbank ID:LC436362.1）
MVHWNHPPTFVLRNICDITGRVFELKENPRIAEANSAVLKWFEQFNVYDKKKADKFL
NVGKFDIFAALSFPEADLEHLTTCLIFFLWAFATDDLSDEGEFQSQPDQVQHGHDISCSI
LDDDDAPQPDYPYAAMLWDLLRRLRSTGHMGMYKRFKQAFLDFSSSQVQQSTNRNV
DRIPPVDEFILMRRKTIGAALVEAMVEYSLDLDIPSYVWEHPVIVGMSQATSDIMTWPN
DLCSFNKEQADGDYQNLVCVLQHNHGLELQEAIDLLTKMISDRVQDYVDLKNQLPSF
GPDVDPALHTYLTALEQFVQGTVVWYYSSPRYFRHLDPRGKPEVLIHLFPKTDAPTLPV
VVQEKSRPFIEREIYLPAKRLLGVFVNYVAITVFGYSVYRLYGDS
>Hfas94a（Genbank ID:MK287936.1）
MSAQQYIIPDLLANWPWQRVSNSMLDEVRDEANEWVMSLGLFEPAQFKKFRACDFN
LLASFIGPLESKEHLRVACDLMNFYFAFDEYTDVASREEAMKIAQGVMDAFKTRSAEPS
SSKITEMARQFFRRTVDVVGEDSPAINQFITDFDTYTTAVIQEADDRAEGTIRNVADYFT
LRRETCGAKPSFSFFALGLNMPTEVFEHPLIMSTVECATDLIAIVNDMHSYGLERARGL
DGHNVVTSIMYEHQLDLQGALYWLAGYAEDTIAKFFSEKERLPSWGRAVDLSVQEFV
DRLGRCVRGYDAWSYETNRYYGNCGVQIRQTRRITLPGNDSGYITKKEMGVSIA
>Hfas94b（Genbank ID:MK287937.1）
MSYAPTVEELKQWEKGPNPELLFGNSFPLARDHPDWQKLEQENDQWVEENWQFDD
KELREYLMSSKLGPFSSMCFPFGEREKLLWICRLVTLLFCLDEDLDRHVNLHLLPILKAL
TAGSRKPENNYAELAIDKCWRAIERTSTPSTFRQFVQITHEYFDSHGQIPYENFDQYVG
ARRTNVGAYFMWACLRYAAGIDLTDEELAHPLIKRLEDIAGFHIAFTNDLISYTKEFLT
DTATNNILTLLQRNDGLTPAEAEAKIRRELKKSEDDYQVAAKEVLNHPVLGKNKAIRK
LVLNIPYGMGGNAWWSLITKRYNVDPVNHPLPQVKIHIDPSMPYHPDAVAFLDNGSK
KALTTKGAKSFLSSWTVWFGALL
>Hfas255
This sequence is not available.
>Hfas344 (Genbank ID:MK287938.1)
MSQSQFTIPDLLASWPRAKNPALDQNLEDEANAWVASLELFEPRQLDKFKACQFNLL
ASLVGPIEGRDSLRISCDLMNFYFAFDEYTDVVSGDEVMMIVADVIQAFRDRESPEGSSK
IKEMARQFFQRTIALVGEDTQGIDHFIADFEAYAKSVVQEADDRVQGIVRNVEEYFILR
RDTCGGKPSFSFFGLGLCIPKEVFDHPVMQSLTESATDLIAMINDMHSYALEHARGLD
GHNVITAIMHEHSVDLQGAFYWLSGHASKTVSKFLNDRKNLPSWGSDIDKAVNEYID
RMARCVRGYDAWSYETNRYYGKNGLEVQKSRKIMLQHRELEMGYITRDQLIGAA
>Hypsu1_138665（JGI ID: Hypsu1_138665）
MALSNSNSTTATVTLPDTLRFWPWQRHINPHYSACKKASSEWCESFKAFSPQAQRAF
NKCDFNGCRIGCDLMNLFFIIDEHTDIASAETARTQANIIMEAIRDPEMPRSENEWVG
GKAAQQFWLNATKSATPSAHRRFIDAFQMYMDAVVQQAADRSKNYVRDIDDYFVVR
RDTIGAKPSFAICELYLNLPDSVMEHPVIMKLTELCIDVIIIGNDLCSYKVEHEHGDDGH
NLITVVMNQFKITPQEAMNYISDLHDKLAVQFLDEWKNIPTFGGPLDLEVRTYCHGLG
NWVRANDSWSFESERYFGKRGIEIQTTRQIEMNIQSHL
>Agr1（JGI ID: MN146024）
MCASATRPQPSASNNVKKIILPDLVSHCTFKLRHNRHRKQVTTETKKWLFKDGNLLG
QKERAYHGLKCGLLTSMCYPDAGYPQLRVVNDFLTYLFHLDNLSDEMDNRGTTTTAD
EVLNSLYHPHTWRSSARVGKMTRDFYKRLVLTASPGAQQRFIETFDFFFQSVTQQALDR
ASGVIPDLESYISLRRDTSGCKPCWAMIEYANNLDIPDEVMDHPIIRSLGEATNDLVTWS

NDIFSYSVEQSKGHTHNMIPVVMYQEGLDLQAAVDFVGDMCRQSINRFVEEKARLPS
WGPKIDQDVAIYVQGLADWIVGSLHWSFETERYFGKSGRQVKASRIVDLLPRQRLP
>Agr2（JGI ID: MN146025）
MVWDFVLSLFHSLLAAFQTLTSWLTGSFLFNNKMAPAPNPAPVTFILPDLEKTFNSLPD
DGLNPHHDVACAESREWFAKYNKKVLGAQMQEFFRRCKFELITSYTYPYVDKEGLRA
TMDWHNILWFFDEVTDTETGKDAHKSAIITIRTLREPDFDDGSSLCRMVRDFRLSHLSR
AGPECTRRFLEHCDVAFHAGAVEAELREKGEVLSIEGYLKLRRETSGARTCFDMAEYL
MDIDLPQDMYDDPVFQKGYIAALDLIFLANDLYSYNMEQAKGHNGANVLTVVMKET
KLNLQSAADYVGVLCEKLIKQFQEAKSTLENRLAKEKNPAKAAALKDAIRSLVGYGH
WVRGNVEWSFETERYFGKKNKEIKKSRVVTLTPTNSVNRALKA
>Agr3（JGI ID: MN146026）
MNASPFLNESSPTRPTSFVLPDLVSHCKFPLSYHPNGDEIAQESVDWLDSSCPDLTAKQ
RRALRVLQSGELTAYCYNQATSPERLRVVSDFLTYLFHLDNISDGMMTRETDVLADVV
MNAFWFTDKYMPTRGPGKEQLDEELNPGKLARDFWSRAIADCGVGVQARFKETMG
LFFEAVNIQARMRDEDTIPDLESYIDVRRDTSGCKPSWVLIEYALGIDLPDHVVDHPIM
QALNQGTNDLVTWSNDIFSYNVEQSRGDTHNMIVILMEYHGHTLQSAVDYVGELCA
QTIDTFCENKERLPSWGPEIDDMVARYVKGLQDWIVGSLHWSFQTQRYFGKDGLDIK
KHRFVKLLPLEAAK
>Agr4（JGI ID: MN146027）
MSALPSQFKLPDLLSTCPLKDGTNPAYKKAAAESRAWIGSYNMFADRKRAFFIQGQNE
LLCSHVYCYAGYEQLRTTCDFVNLLFVVDEVSDEQSGEDARATGQVFVNAMKYADW
HDGSKLAKLTKDFRVRFLRLAGPKNVARFVALCESYTACVGKEAELRESGQVLGVKEFI
PLRRQNSAVLLCFSLVEYILGIDLDDEVYRDENFLNAYWAACDHVCWANDVYSYDME
QSKGLSNNNIVTVLMEENHTSLQDTSDYIGEKCAEFVQIYLTSKKRLSPSLGPDAALFLE
SIGSWMVGNLAWSFETSRYFGSRHLEVKETGIVILRPRELPEDGSSSDSDEE
>Agr5（JGI ID: MN146028）
MASSLLEPSLAAIALVILLASVSLSRKKRPAAPEPQGLSVLGNLFDIPKRASSIIYLALGKP
YNTLTKRAVSQLQGYTPGSHIDATSHSPRVFRLPNLEETFSVFPDHGLNPNYTSARTDS
RAWINQYTKVVCGPKMVAFMNNCEFELSNSHCYPYAGYKGLKATMDLTNILWLYDE
YTDTGSGAEAVKAAGIVARALREPDYDDGTWVCRMMKSFKQNHIDKAGPGVARRFI
DNFCNYVEVVGREAELREKNEVLDIPNYVTFRRETSAVRTCFDLVEYCLDLDLPQYVH
DDPVFISGYNAGMDLVFWANDLVSYNMEQSKGHSGANVVTVIMKSKGVDLQTAVDF
LGGYCEALTAQLLEAKRILQARSDAAYSRDVVRLMDAFGDWVRGNVAWSFETERYFG
KENKRVKETLLVELKEPFVGALALKE
>Agr6（JGI ID: MN146029）
MPGSANWTADRFYIPDTLANWPWPRAINPAYEECKAASAAWCEKYGAFSARAQKAF
NLCDFNLLASLAYAGLPADVNRVGCDLMNLFFVVDEHTDAMDARSVQDWVDIVVD
ALHHPHTPRPAGEPKVGEIARTFWENGIKCMGPTAQRRFVETFTTYLQSVVTQAQDR
DKHLFRDVDSYMEVRRDTIGAKPSFALLEHDMELPDDVFYHPLLEKLREWAIDMLILG
NDLCSYNVEQSRGDDGHNIIRLAMLQENTNVHGALRFVSKMHDDLAEKFLSNYQG
MPSFTPQIDAWVTRYIDGLGNWVRANDSWSFESWRYFKGDVLRVQAERWVELLPPAP
KDELTPA
>Agr7（JGI ID: MN146030）
MSFFKSSQPTIYIPDTLRNWPWPREINPHYEECKRESAAWVEKFGAFSAKAQKAFNKC

DFNLLASLAWSRVNRDGCRIGCDLMNLFFVFDEWSDVSDAEETRRMADIIMDALYDP
HKPRPTGEWVGGEVTRQYWLNAIRTATPSAQKRFIKAFKLYTDSVVQQSADRDKHLIR
DIDSYFEVRRDTIGAKPSFAINEVHMNLPDYVMEHPVIKNLTAYCIDMLCIGNDLCSYN
VEQSRGDDGHNLVTIVMHQLNLDVQGAFDWIGKLHDELVDKFLEEYKNVPTFKDKQ
VTKECAEYAFGLGNWVRGNDQWSFESERYFRKDGMRVLTERTVVLLPKKREPPPKPL
DEDPIYSALPWWGWTALFGFLATAFTFSARQLSTRISANLIA

>Agr8 （JGI ID: MN146031）

MSEQQYTLPDLLQNWPWNRHLSPYYEEAKRESSAWVESFKPFDQDGQRAFDAYLLAS
LTYSHGSREFVRLGCDLMNFYFVYDEYTDVSDSAVADRLANIVIDAMRNPENSSQSGD
HLLGKMTKHFWTRALAMAPAGSPCFEHFITTSETYLRAVTQEAEDRANKRVRKVDDY
LRLRRDTCGARPTLALIEFGLNLPNEVVRHPSLVALTEAAVDLIILVNDMHSYVRELSC
GHENHNLITAIMLEHRLNRQDAFHWLGSHCSRVVDQFLSDLDELPSWGEPTDSGVRD
YINGLGQWVRGNDDWSTESKRYYGEDGETIRQERLVTTRSGESNYIKFGQVGVQDSVR
IQPIEAN

>Agr9 （JGI ID: MN146032）

MTAAPLTFTLPDLLANFPWKRNLSEYYPECKTESSAWTESFHPFDDEGLKGFNLCDFN
LLASLAYSPREREIIRLGCDLMNIFYVFDEYTDIADGDGADKIRDIIMDAFRNPHKPRPE
GELLVGEMARDFWIRASGYVSPDAHCLTHFLRDFDTYTAAVVREADDRAKRVYRTFE
DYLSIRRDSSGCLPSFALCEFGLDLPEEAYHHPRMAALREQSTDLIAIGNDIDSYAMEKA
RGLELHNSVELIINEHGLDVQGAINWLERYAAGVHASFLDNVANMPSWGEDVDRRV
KMYIDGLAQWVRGNDDWTFESGRYFGDKGLEVQKTRVMSLLPASKVSLRSRPKAVG
HVPKKLLRYFRYSTMYFFGFHVLAK

>Agr10 （JGI ID: MN146033）

MSLNFFLRRYTVFPWSRKLGQYYHDAKRESSAWTESFHPFDEDL
SKAALTFCITALLASLAYFLRQKEIVRLGCDLMNIFYVFDECADIADKEGASQIRDVVM
DDLHRPEKTCPGGEILPGEMVKYVLLLCPEIPETYYNTKSFGFAPQSSSPQPHIVCATLS
RISMPTQQQWFNVKRTIGPNVFLARSATVLPYAETHTFYHPRMIALREQAPFLDINSYP
MKVRGLVQGSINWLEGYAAGVQAAAFLDNIANLPSCAKEVESRVNIYVNELAQWAR
GNDDWTFESGRYFGDRGPENQSDIPTSSNR

>Agr11 （JGI ID: MN146034）

MQIILPDILQTWAYARLLNPHYDGAKLESSLWIHPLVAKLFDQK
GQKAFQNDYTSLLASLMYSHQGKVPSRRCDMMNLFFVYDEYTDVVSPEIAHRLSKIV
VDAMKNSDEMSPCGEHPIGDKAKEFWRLATTLLPATGSNSDVCKSRFINLTEEYLNAV
TVEARDRNEGTIHSVKEYLTMRRATSGAGLMLALIEFELDLPKAVLEHKFVQALEEIYT
RTVSSGQANHNLITVVMHENPGLSLQGAFDWLGSYAAGVVECFQTNVRNLPSFCDVE
GPACESVDGTLQERVDKYISGLGQAVRAEDDWAFETTRYYGEDGPKVRETRVLVIRPV
KRITRRHLLQSLEIKYSMVRG

>Pro1 （JGI ID: AGR34199）

MSQRIFLPDTLANWQWPRHLNPHYAEVKKASAAWAKSFRAFQTKAQEAFDRCDFNL
LASFAYPLADEARLRSGCDLMNLFFVIDEYSDVSTEEEVRAQKDIVMDAIRNTEKPRPA
GEWIGGEVSRQFWDLAKKTASTQAQKRFIDTFDEYLESVVQQAADRNNSHVRGIESYL
EVRRNTIGAKPSFALLEFDMQLPDEVINHPVIKELEKSCIDMLCLGNDVVSYNLEQAR
DDDGHNIVTIAMNELRTDVAGAMIWVDEYHKQLESRFMENFKKVPRWGGPIDLQVA
RYCDGLGNWVRANDQWSFESERYFGKKGPEIIQRRWITLMPKMVSEELGPQIVDGFH

L
>Galma_104215 (JGI ID: Galma_104215)
MNTTTRTFYLPRLEDTFSVFPDNGLNPHYAECRIQSQAWIDKYYKIVCGPKMRAYMD
HCKFELITAYTYPYASSDGLRKTMDLANILWLYDEFTDTLSGKDATNAAAIVIRTLRERD
FDDGSWICHMMRDFYAAHIEKFGPNVSRRFIDHFCQYVEGTGTEAKHREKDHVLDIN
AYIIMRRAASAVLTAFDLAEYCLGIDLPQYVHDDPAFISGYNAGLDLVFLDNDLFSYDM
EQAKGHCTTNIITVVMKSKRIDLQSAFDFTAGYCESLTQQLIAAQISLASRTDPVFSNN
AVKCLEAIANWVKGSDGWSFATERYFGKQNVIVKETRAVEMRKSFQDIAVLKE
>Omp1(JGI ID: 1311)
MANPYAVWNAGQSGTWHGQAVNFDPPPSIFGALPFVSRVEEFPALTFSFTAYNPSILNC
IVLGPNNSPQFRVVTDPAMPGYTLLKTMDERSSALVEWQQPPKIEIRGAVLKQPTSNFL
RLSPNRRYRIMVIGDQEFMWLPDQSSVCVSLSTFDAMPLARISRNGNSISLEVTQTAIRA
GLLNTCVVTTVLLQCGRSID
>Omp3(JGI ID: 4636)
MAIENTIASAPASTPAKQLDTPDHFILPDLVSHCTFPLVYHSNGDAVAAQSVKWLDTN
CPDLNDKRRKALYGLQAGELTAYCYNTAPDQRLRVVSDFMNYLFHLDNISDGMMTK
DTDALSDAVMNALWFTEWYRPTKKSDYVQPDEELNAGKLARDFWHRCIQDAGPGC
QARFKETLELFFEAVNIQAKARDAGVIPDLESYIDVRRDTSGCKPCWALIEYGLGIDLPD
YVAEDPIIKSLNQSTNDLVTWSNDIFSYNVEQSRGDTHNMIVILMLYHGHNLQSAIDYV
GDLCRQTIDDFKENRKKIPSWGPEVDDIVKQYVQGLQDWIVGSLHWSFMTTRYFGKQ
GQEVKKNRYVKLLPVGEEANKW
>Omp4(JGI ID: 1447)
MYIFCSQVVSGLNKFTFKMSSAPTRFLLPDLLSACPLKGSVNPYYKEAGAESSAWINSY
DIFTDRKRAFFVQGCNELLVAHTYPYAGYEEFRTCCDFINVLFVLDEVSDEQSGSDARF
TGEVFLNALRNPENDDTSKLSKISKEFRARYFKRAGPRTAERFLQHCQDYIDCVTREAE
LRERGEVLDLPSFTALRRENSAIRICFCLFEYALGFDLPQEVFDDPTFMEMYWAAADLV
CWANDVYSYNKEQAQGHGGNNIVTVLMKAKDLDLQAACDYIGVYCEELMGRYLSA
KARLPSWGPEVDAAVAQYVEASGHWVRGNLDWSFETQRYFGAQHAEIKETRLVTLTP
AIPEDFSDTGSESE
>Omp5a(JGI ID: 2392)
MSPDPTRIVLPDFLAACPFESSTKNPHLKAAGAESSAWVNSHVVFNDRKRAAFMQDI
YELLVAYAFPWADYEDFRTMCDFINLLFVLDELSDDQNGKDAGYTGKLFMDAMRNID
NGDTSELTELCREFKARYSKRVSPQVNERFLQHLQSYTDCVAQEADLRERGEILDLESY
VALRRENSAIRPCFDLVEYIIDFDIPQEVIDHPVFSEMYWASVDLVCWSNDVYSYNVEQ
AKGHGGSNVVTVLMKEKNLDLQAACDYVGVYYEELMDRYLSAKARLPSWGPEIDA
AVGKYILAEAQFVRGNLDWSFDSPRYFGPQHDQVKKTGIVTLTPAPKKFGSDSGSESE
>Omp5b(JGI ID: 2393)
MSPAPSRIVLPDFFASCPFESSTINPHFKAAGAESSAWVNSHVVFNDRKRAALMQNSY
ELLVAYAFPWASYEDFRTLCDFINLLFVFDEVSDDQNGKDAGYTSKIFMDAMRNIDNG
DHSELLCKEFKARFSRRLSPQVNERFLQHLQSYTDCVAQEADLRERGEILDLESYVILRR
ENSAVRPCFDLVEYIMDFDIPQEVLDHPVFSEMYWASVDLVCWSNDVYSYNVEQAKG
HRGSNVVTVLMNEKNLDLQAACDYVGVYYQELMDRYLSAKARLPSWGPEIDAAVG
KYVLAEAQFVRGNLDWSFDTPRYFGPQRDQIKKSRIVTLTPAPKKFGSDSGSESE
>Omp6(JGI ID: 4774)

MIAKNSEIDRFYIPDTLANWPWPRHLNPAYPEAKKASAAWLRSFNAFNERSQKAFDL
CDFNLLASLAFPLADLYCLRSGCDLMNCFFIFDEYSDVADPQTVRQQADIIMDAIRNP
HVPRPRGEFIGGEAHRQFWERAMQGATPTAQRRFIDTYQQYTDAVVQQATDRADNH
IRDVEGYFTVRRDTIGAKPSFTLLEFTMDIPDEVMGHPVIKDLSLWCIDMLIIGNDLCSY
NVEQAHGDDLHNLVTIVMNQYNLDLPGAMEWIGKFHDDIADKFLDTFAKLPSWGPE
IDPQIRRYVDGLGNWVRGNDSWSFESWRYFRGKGPEIEKTRWVDLMPTEEATITPKYE
SDSNA
>Omp7(JGI ID: 2271)
MPETFYLPDCLANWKWKRALNPNYPEVKAASSEWLRSFKAFPPKAQEAYDRCDFNL
LASLAYPLADKDGLRTGCDLMNMFFVFDEYSDVAHESEVQVQADIIMDALRNPHKPR
PVGEWVGGEVTRQFWELAIKTASPQSQKRFIETFDTYTKSVVQQAADRTQHYVRTVDE
YLEVRRDTIGAKPSFAILELTMDIPDEVIHHPTIERLAILAIDMILLGNDTASYNYEQARG
DDNHNMVTIVMHQYKTDIQGALSWIEKYHKELEEEFMQLYNSLPKWGGQIDVDIAR
YVDGLGNWVRASDQWGFESERYFGTKAPEIQKTRWVTLMPKKRAEGVGPEIVDISEL
>Omp8
MKGEPQEDLFLDALARILLDAPRYYGRLATNIIVTATLDFFTGLFLELQARDMTFNEDL
HNFAVFCRNLTGIAHAYAVFMFPRDVPFTVYVRSLPELKTDINYVNDIMSFYKDRAEET
DNLASILRQVHPSMTKHQVLQKIVDDAVEADVRARKILADYQPALDAYEHFRKGYAM
FHVSSGRYRLDELFSYIRFE
>Omp9(JGI ID: 3258)
MSQILHLLWSKFSTSLPSTVTIGSDPQTLQLVHSPAPNVNANALEIYKIVDNFLSRCGIRL
ESTPLDVEFYNECKKTLLSHYIGIHDSDKVSESWFKRYLSVGVIITTNAYGHLDNKLTKI
YIALYTALATCFDDVFEKNVDHMSGFNERFMKALPQGDVFLDAFAKVLLDAPRYFGR
LASNIIVTSTLDFITSMSVDVLTKGMKFNQNLHNFAMACRNMSGIAYTYAPFIFPKEVP
FAIYAQCLPDMRIYINHVNDVLSFYKEDKAGETENLASILGQVHPSMTKYQIVQGLAD
DAAEADLRVRTVLSQYQPALDAYNCFRQGYVSFHASSGRYRLDELFSFVEPEPIV
>Omp10(JGI ID: 3981)
MRNFLSQCQIPLQRGVPLDPTFHQECANVLIEDYLKPSAAVTLENLPSLMSSFNPFLTL
GVRMASTGYAHLTHTPTRVYVALFTALLVCLDDIFPENVELMCGFNERFIKNETQGEPI
LDAVAGLLRSTSKYFSMLSSNLIVTSALNYVTSLSLDQGLHSIKATEHSRNFARLCRNMS
GIPEAFAAFVFPPEVPFTAYIQCFPDLYTYANYVNDVLSFYKEDIAGETENLVSILAQTQP
NSSRYQVLQQLADEAAAANANIRDILSDQKSILDAYDAFRVGFVQFHIDSPRYRLAELF
PCIDG
>GME9210(Genbank ID:KX281945.1)
MPLSSSVVAFRLPDTLGCWPWRRCLNTHYVEAKQDSASWLESFHPFGPKAQRAFNKC
DFTNCVQGVTTSSSSSTSTLMSRTRRRSSSSRTSSWTHCETLTSLVLQENLSWAKSLGSW
ARTIKVASEPSQRRFIETFDDYCQSVVQQAADRSQNHLRDVESYLENRRENIGAKPSFA
LLELDMNLPDEVIEHPTIVNLTTWAIDMIILGNHCLVQRGAGAWRRRTQRRDHRHAS
LQRRRAGCHGPHRRVAPEAGGPVPHQLQQAAVVGTRDRASAVHPGHRKLGTRERR
MEFREREVLWIERTRDRAESLGNPTPSGLRGEACGC
>GME3634(Genbank ID: KX281943.1)
MAVTPANVASPDSQEIVLKFPDFISPIPYPLRCHVQEREVSRQSEEWLLSMANFSEKQRS
KFLTLNGGLLSGMCYIDCTFDELRVCTDFMNFLFTLDDWTDEFDTTGTRGLAECVMN
TLYWPHSYQADTAAHRLTKSFWVRMKQTAGPGCQQRLMSTLDTYFQAIMQQAADR

GSHNIPELEEYILLRRDTSGCKIGFAFIEYAANIDLPDDVIEHPIIKAMADATNDLVSWA
NCPFVQRTVTRRHAQPRLCSHGSERPRPAGCDRAGGRAMGEDPPLVLRVPQERPFVG
LRDRPCRRSVHPGARRLDYRERRVELRDGALLWQGRASREEDAAGYTASGPYSC
>GME3638(Genbank ID: KX281944.1)
MRARSFILPDLVSDCPYTLRCNSNCEAVARASEAWMLEDANLSPKRRDAFLRLRGGEL
TAACYPDTDEACLRVAADFLNFLFSLDDWSDEFSMEDTCGLAQCVMCVLHDPDDFQ
TEKAAGKLAKLFQSVPADGGAEVYSSIHRYGPLLSCDCTASPGPRVRVCSLTRRICGPPR
GHERVQALLRSHRIRRGYGPSRPRRPSSNNHRPRAGSQRVYIMVERSLLVQRRASAGH
TQHDCGDHARGRAQLARSRILGRSLQALHSALRGKPSHAAIVGSGD*RGSRQVCARP
PGLDGRCPPLELRHRTLFRGRPCDQEARCRDTTTAEVFI
>Cun3817
MVMYKRNATFNDGPVTQIQDIPSISSLFFTMVNQLRPFPSRFVLRDLTAVTEPVFKLRTN
PHQEEAYRNIERWFKDLKVYPEPKQRKELSHAEDLYAGLSFPDADVEHLETCIAFFLWA
FSFDDLSDEGEFQSKPDRVQVGVDISMEVLNHPELPPPKFKYAAMLHDVWRRFRSTAS
PGACDRFKAVESWMKSQVEQAANRSWNTVPSVDEFILLRRRTIGGEIVEAMVEYSLDI
KIPEYVWDHPVLVGLSRAAIDIMIWNDLCSFNKEQADNDFQNLVFCVMLERNVDLQS
ALDIVTKMLATRVDDYAKFKAQLPSEGAEVDQELAAYEKALEHYVQGTVWYYESPRY
FRGMDVTDKTDMVIPVYSRSADAPTSPSVSTRVSYLSNRSMPPRSSKASV
>Cun5155
MANESLTTTQLFIPDVLRSWPWPRQINPHYAICKAESDTWAQSFNAFSMKAQQAFLR
CDFDLLASLGCPRLDKEGCRICCDLMQLFFIFDEHSDIVDTTVVRRQADSIMAAIRDPT
RPREGEWIGGEISRQETANAMRVSTPTFQERFIVAFQEYTDSVVQQALDRDRHNIPNIE
QYFNYRRDTIGVKPSVAMLEIQFDIPNEVSNHPAISTLRSTCVDMIAINDLFSYNVEQAL
GDDEHNLTTVVMHEHECSLTDALEWISDLHDSIANTFLSVMKTVPSFGDLVIDEQVAI
YVDGLGNNVRANEANSFESERYFGKNGLEIQESRVVDLLPKQEN
>Cun3157
MSSNAVSFTLPDLLAICPLQGRTNPHYEAAAAESSANVLSENVFSNRKQDFFVSGGSEL
LCSHAYPYAGHEELRICCDEVNLLFTYDEISDEQNGQDAYKIGLVLLKSLRDPEYNDGS
VLCTMTKQFQERLFPRMGPACYERFVDHVENYINAFVKHAEYREKNVVLDMASYEIL
RRLNSAVRCCFGLFGYVLGLDLPDEIFEHPDMMAMHLAAVDMVCWNDIYSYNMEQ
AMGHTTNNIMTVLMKAKNVDLQGAADYVGEYFKVLMDRFFDHKSKLPFFGPDMDP
TAEQFVMAMESWIVGNLANSFETLRYFGEKREQVKVTLVVELASKKV
>Cun3158
MPALTRTFKLPDLLSMCPVKGSTNPHYEKAAAESSANINSYNLETDQKRAFFIQGSNEL
LVSHTYPYADYEQERICCDEVNLLFVYDEVSDDQSGRDARATGNVFLQVMRHDDWD
NGSPLAQMTKEFKARFRKFAGPGCYARFLVHCENYINAVGHEAEYRERGYVLDMESY
ETLRRLNSAIRLCFGLFEFVLGVDLPDEVFDDPAFLNLYNAAADMVCWNDVYSYNME
QAKGHSGNNILTVLMQAKNIDLQTASDLVGDHFAELMRRFLEGKRALPSNGLATDTA
VAAYVKALEHNVKGNLVNSFETQRYFGPKHEEIKKTLLVVLRAPSFD
>Cun0773
MVAHTFSLPDFFAKTPYQSRLHPLTGIVVQKSQEWVLRKVNYDEKERTAFLKTSGGLL
CGYCYPNADAFHIQVCADRMDWVFCLDDWSDECSVAEAQSVINSIKEYPRYPHEHSG
STPIIELAKNLYDRFFQTAAPGCAERFVRSMDIYLDGVVEQADCREKGSILDTESYTILRR
KTSGVWPCFALIEFAARIDLPDAVVEHPLIRSMEEATNDWISWNDILSYSKEQADKDA

HNLIAVIMNQYTLDLQSAVDLAGSHCFDCITKFEDNRKALPSWGEEIDREVDLYVQGL
QDWIIGSLHWSFACRRYFGAEGKEIKEHRTVFLLSEKHQS
>Cun7050
MQSATAPVLSSQPSKVVIPDLVSHCDFTLRCNSNQERASAECKEWLFQGSNLSERKQN
AEHGLKAGLLTSWCYPDAEYHHLRVCCDEVNWLFHLDNISDDMNTENGTSRTAVDI
MNTLYHPYSYSPVSPEGKLIQDFWRRLIPTASPGSQQRFNRTMDFFFQAVTQQALDRA
NGVVPDLDSYIALRRDTSGCKPCWALIEFAYNLDLPNEVMEHPTIVALGEATNDLVTW
NDIFSYNVEQSKGDTHNMIVVMMRQEGLDLQSAVDFVGDLCKQSIDRFIEQRAHLPS
WGPDIDQQVEKYVGGLADWIVGSLHWSFESERYFGKSGLDVKKTRVVELLPRRA
>Cun0716
MSRFNADSFVLPDLVSHCTYPLNLNTNWHSVSRASEQEVLEEANFSEMKRGVFMGLK
AGELTSACYPYCDAFHLQVAADFLGYLFTLDDWSDDFDELGTKGLAVCVMNALRDP
HGFQTDKPAGILAKDFFSRYISKGGPGCIERFIDTMDLFFIAVERQAIDRENGVVPELEA
YIPTRRDTSGCKPCFALIEFAADIDLPDEVVEHPTIAALEEATNDLITWNDIFSFNVEQSR
GDTHNMIIVAMRERGLDLQDAVDFVGELCKQSIDRFEHDRLTVPSWGPEIDRDVRTYI
QGLQDWIVGSLHNWSFDTTRYFGQDGTEVKLHRQIKLLPRKGPCSD
>Cun0759
MARSTEALPDLVSHCPYPLRINPLCDIVTQKSEEWILNEAKYTPEKRIRFLNTKAGILTA
YCYPDADDFHLQVSSDYLTWLFCFDDWSDEFDETDACSFADCIMGCLRDPYGFKTDK
AAGRLTKSYFGRYLQTSGPRCAERFIDTMDLYLKSVAQQAADRDDGRTPDLETYIGLR
RDTSACRPCFALMEFVAGIDLPDEVAEHPLIRSMEDATNDLVSWNDIFSYNKEQSRGDT
HNLVAVIMEERKLDLQSAMDFAGELCHQSISKFEADRRSLPSNGKEIDRDVQLYVQGL
QDWIVGSLHWSFATKRYFGTEGEAVKHHRTIQLLPRKDGVEPKDTVVRQNVIVYLFN
MFFTVLVGSLFGNMKEGLSSPLRVPRP
>Cun3574
MSPKRFVFPDLVSHCPYQLRIHPDCDTVNKTSEEWIMKDITFTPDRLKRFLDIKAGVLT
AYCYCDADLFRLQASSDYLTWLFCYDDWTDEFDEDESCSFKDCILGCLRDPYGYKTDK
IAGRLIADFFRRELQTSGPHCAQRFIDTMDLYLDSVGRQAADRIEERTPDLESYIALRRD
TSACKTCFALMEFANGLDLPDEVSEHPLIREMEDATNDLVSWNDIFSYDKEILIEDTHN
IVAVIMEDKKLNLQSAVDYAGDLCNNCIARFDEARKQLPSWGPEIDREVQLYGQGLQ
DWIVGSLHWSFVSKRYFGLEGEAIKKHRTIELTPAMKSDVEA
>Cun5765
no function
>Cun6114
no function
>Cun7487
no function
>Cun0802
no function
>Cun9106
no function
>PpSTS01(JGI ID: Pospl1_60326)
MSSSAPSTSAPTKIVIPDLVSHCTIPVRCNRHWKQASVESKRWLFRGGNLSDRKRDAFH
GLKAGYLTSMCYPLAGYPQLRVSCDFMNYLFHLDNISDEMNDRGTHGTAVSVLDALY

QPHMHPTSRVGKMTKDYWVRLIQTASPGAQQRFIETFDMFFQAVTQQAMDRANGVI
PDLESYIAIRRDTSGCKPCWALIEYANNLDLPWEIMDHPIIRGLGEAANDLVTWSNDIF
SYNVEQSKGDTHNMIVVVQNQQGLDLQSAVNFVGDLCKQSIDRFHYLRENLPSWGP
ELDREVEIYVDGLADWITGSLKWSFESERYFGKAGLEVKKTRVVALLPRRA
>PpSTS03(JGI ID: Pospl1_99496)
MGSISSTPSQKSPVFPTPSLLPSDIVAVRPEGDEAKVLKFPDLVKSIPFPLRLNPYIRFVSAE
SDAFIIEYANFSEKQRNRFIGLNAGLLCGMCYAECGPEQLRVCCDFMSFLFNLDDWSD
EFDTAGTKGLEEAVMNTLYHPDTYVSDTVAARTARSWWTRMLKTVGPRCRQRFVETL
GFYFKAILQQAADRSSKTIPDLETYISLRRDTSGCKTGFALIEYAAGIDLPNEVVDHPIIQ
SLLDATNDCVSWANDILSYNREQSRGDTHNLVPVIMQTVGIDRQAAIDYAGDLCNKS
VAHFLEGKAALPSWGKEVDVQVEQYVQGLEDWIIANAEWSFMTERYFGKDGPKIRK
GLQVSLLPVVGFD
>PpSTS06(JGI ID: Pospl1_45581)
MTVIADTSRCFILPDLISYCQFPLRCNPHRDAAQSSTSWLINNYPGMSPEQLVEVRRLD
ADTLASYCYPDCDVERLRVASDFLAILFHLDDITDTMEEGGTEQLEGTIMDAFRSEGKL
DQREDEPRVRVPAKDLWTRFIRNAKPCVQTRLRDNIALFFKTAREEARDRERGVLLDL
ESYINMRRGTSACLSCFALTEYSIGIELPQYVVDDPIVQALNQSANDLVSWSNDIYSFNN
EQAHGIHNMIVILMKSQGLGMQDAIDYVSDLFKQTIDGFMENTQLLPSWGAAVDAD
VRLYVQGLQDWVVGNLHWSFATERYFGKRGAEIKATRVVELLPKKPVS
>PpSTS08(JGI ID: Pospl1_59374)
MLYLPDTMSAWPWQRAINPYFNEVKAASNSWFKSFRAFSPASQKAFDKCDFCLLAAL
AYPRARKEHLRTGCDLMNLFFVIDEYTDVEDANVCRDMVDIVIDALRRPHDPRPEGE
VVLGEIARQFWARAIETASPTSQRRFLETFIAYLESVVLQAADRDCDAEHTVQTYLAQR
RDNIGSYPSYAVLELALDIPDDIFYHPAMNELSLYATEMLIIDNDLVSYNREQASGDTNN
ILFVIMRQFNCSLDHAMAWAAAYHSQLEARFMDAFKRMPSWGLEIDSQVEEYCQGIA
NWPRGNDCWSFESGRYFGDKGREVQKTRCVPLLPKKERDTSLRQQDVVITSL
>PpSTS10(JGI ID: Pospl1_98072)
MPSTPRQFVLPDLFPLVPFKGSTNPHYVKAAAESSAWINSYNVFTDRKRAFFIQGSNEL
LVSHTYPYAGYEQFRTCCDFVNLLFVVDEVSDEQNGKDARHTGNVYLKAMRDPEWN
DGSVLAKMTKEFRARLLQYAGPGCYARFLKHCEDYVEAVAKEAEYRECGVVLDMASF
ETLRRENSAIRLCFGLFEYCLGVDLPEYVFEDPTFMTLYWAAADMVCWSNDVYSYNM
EQAKGIGGNNIVTVLMQAKGIDVQAACDAVGEHCKLLMERYLDAKEKLPSWGPSVD
DAVAGYVQAMEHWIIGNLEWSFETQRYFGAVHAEVKATRVVMLRPREIDED
>PpSTS14(JGI ID: Pospl1_101549)
MSDQPKMIYLPETMANWPWPRYINPHYEEVKAESDAWFKGFKPFTKQSQVAFDKCD
FGRLASLAYPWASKEHLRTGCDLMNVFFMIDEYTDVECASVVRGMVDIVIDVINNPH
KPRPEGESLLGEITRQFWERAIKAATPSSQKHFIEAFTDYLNSVVEQAADRDNNHIRTV
DSYLKTRRENIGARPSYFPAELGLNLPDEAFYHPVVTELSYNIAELIILDNDIASYNKEQ
ATGDDRHNILTIVMLQFNIDLEAAMTWVASYHKDVENKFLDGMKKLPSFGPVVDKEL
EEYILALAIWPRTNDCWNFESGRYFGSKGLQVQKTRYVPLLPKVKTDPTLKQKQVVVS
LVDL
>Fompi1(JGI ID: 84944)
MSRRYQTSRFSPSAKGKKTSLYTSPGDLRALFHRFSLYAGVQLESFTSPKMMQIEVPVML
NLKESSMPMNSTVLEDCTAAVAKDAIVQFLRRLGAVLRPSFGNNRDLEERVKEITKTW

PFEHRIHPHITTGVVMANTTIAYLSDLDARAAVAAYTALITALDDPDIFHASGAQNFAQ
MLCDGSALRDDGVLGQMARVLADMGNHFPPFGTSAIIAATLRWCNGELISNPANPFC
LRPLSKAFADYQRGLTGVPEAYAAFGWCKADFPVETDYIHVFPDICFFLNHTNDILSFY
KETLDGESDSYIHARARLTGKSVTDTLYEVMDEVITTTERIRKHFGEGRMRNAWDRYE
AGYVWFHTGNPRYRLHELVDTEYMPMY
>GL26009
MAVTPASANASDSTKEIILKFPDFITPIPYPLRCHVQERQVSRESEEWLLSMANFSEQQR
AKFLTLNAGLLSGWCYIDCTFDELRVCTDFMNFLFTLDDWTDEFDTTGTRGLAECVM
NTLYFPDTYQSDTAAYRLTKSFWDRMRATAGPGCQQRLMSTLDTYFQAIMQQASDRG
SRNIPDLEEYILLRRDTSGCKTGFTFIEYAANIDLPDEVVEHPIIKAMSDSTNDLVSWAN
DVLSYNAEQSRGDTHNLVCVLMHQNNVDRQEAIEQAGALWEKTLNWYFECRKAVP
SWGPEVDRAVAMYIQGLDDWIIANAEWSFETERYFGKEGHTVKKTRQIALLPQRAHA
>GlSTS6 (Genbank ID: UDP19923)
MSVEQLRPFPTHFRLKDLAAISGRVFEFKLNPHEKEAAKATYAWFDGRNVYHGLKKK
RFLSHRFDSYAGMSFPDADVSHLETCIAFFLWAFSFDDLSDEGALQSKPEAHQVGVDIS
MEVLRNPTAPPPNFPYAAMLHDIWRRFRETASPGACNRFFRAVESWMNSQVEQARN
RATDEIPSVEEFIILRRRTIGGPIVEAMVEYSLDLQIPEHVWDHPILQEMSKAVIDIMTWP
NDLCSFNKEQADGDFQNLVFCIMIERDCDLQTAVDVLTEMLSQRVVDYERYRAQLPSF
GPEVDAELARYNKAMEQYTQGTVVWYYHSPRYFRGQEVTGIPEIVVPVYERTTPAPEE
PTAPASKSARSAPANLAPSSSKEVGLLVNAPTQHNARLSLLLISLVACLLCASFISFPSFPL
NPRLVLSI
>GS14272 (Genbank ID: PIL24516)
MAIAASKPKPTPDHFVLPDLVSHCNFPLAYHPHGDDIAAESVRWLDEGCPELSPRGRK
ALYGLQAGELTATERLRVVSDFMNYLFHLDNISDGMMRKGTEELADSVMNALWFPD
RYMPTACDGKEQPADEVSAGKLARDYWTRCTRDAKPGPQARFKENLELFFEAVYQQ
ARDRDTKLIPDLDSYISVRRDTSGCKPVFDLIEYAMDIDLPEHVVRHPVIQALNQGAND
LVTDIFSYNVEQARGDTHNMIVILMELRGFDLQTSVDFVGELCRQTIDTFMENQQNVP
SFGPRLDRDVALYIQGLQDWIVGSLHWSFMTERYFGKSGAEVKKHRI
>GS11330
MLATLSPFASGVHYPVVPITEETKDPLAAVKVLIRDFLDRSDYRSPGSPCDQELRRKLTE
ELSAWPSDIKPTLVAKIMDGSCVYAETTYAHTTHEHRYFIALYTACLMYVDDLGERNLD
AVKRFTSRFAKGEHQPDPILQRLAELLGRAHDLWTQFGADAIIAGTLDAVTAMYIEFTT
QGMVVKPSATRFPYYLRTRAGLGPPYIHFVFMNDWRATPESYLQVLPSVVHSDIGADS
LLWRLSFYKEELAGETNNYVHLRTSAEQSSAANVLRHLVEEVLESAGTMDMLTSGDSE
LAALWQRYMQGYLEFSLKAKLQSGDA
>GS02363(Genbank ID: PIL35634)
MAVTPASANTQDSTKEIILNFPDFISPIPYPLRCHPQEREVSRESEEWLLSMANFSEQQR
AKFLTLNAGLLSGWCYIDCTFDELRVCTDFMNFLFTLDDWTDEFDTTGTRGLAECVM
NTLYFPETYSSDTAAYRLTKSFWERMRATAGPGCQQRLMSTLDTYFQAIMQQASDRGS
RNIPDLEEYILLRRDTSGCKTGFAFIEYAANIDLPDEVIEHPIIKAMSDSTNDLVSWAND
VLSYNAEQSRGDTHNLVCVLMNQNGVDRQEAIEQAGALWEKTLNWYFECRKAVPS
WGAEVDRAVALYIQGLDDWIIANAEWSFETERYFGKEGHTVKKTRQVALLPQRPRA
>GsSTS43(Genbank ID: PIL26225)
MSVEQLRPFPTHFRLKDLAAISGRVFEFKLNPHEREAAEATHAWFDSRNVYHGLKKK

RFLSHRFDSYAGMSFPDADVSHLETCIAFFLWAFSFDDLSDEGALQSKPEAHQVGVDIS
MEVLRNPDAPPPNFPYAAMLHDIWRRFRATASPGACNRFFHAVESWMNSQVEQARN
RATDEIPSVEEFIILRRRTIGGPIVEAMVEYSLDLHIPEQVWDHPVLQEMSKAVIDIMTW
PNDLCSFNKEQADGDFQNLVFCIMIERDCDLQTAVDVLTEMLSQRVIDYERHRAQLPS
FGPEVDAELARYNKAIEQYTQGTVVWYYHSPRYFRGQEVTGIPEIVVPVYERTTPAPEE
PTPSASKSARSAPANVSPSLSKQAGLFVNTPVQHNARLSLLLVSLVACLLCASFVSFPSF
PLNPRLVLS

>GsSTS45a(Genbank ID: UDP19925)

MSIEQLHPLPSHFRLKDLAAITGRVFELRLNPQEREAATAAHAWFDSHHVYQDLNKH
RFISHAFDSYAGMAFPDADVSHLETCITFFLWAFSFDDLSDEGTFQSNPQAHQAAVDIS
MKVLRNPAAPPPDFPFAAMLHDIWSRFRATASSGACNRPVHFYRLFLLLILNHISRFFR
AVEGWMRSQVEQIRNRAIDEIPSVNDFIILRRQTIGGPIVESMIEYSLDLRIPEDVWDHPI
LQDMSNALIDLMTWPNDLCSFNKEQADGDFQNLVFCIMIEQDCPLQTAVDILTDMLS
QRLVDYEELKAQLPSFGPEVDAELTRYIKAIEHYTQGTVVWYYSSPRYFRGQAVSGIPEI
VVPVYEKSASMQDTTLTPTAKASKPAPVVKHSPWVKADSSFVHTHVVLQCMIYLLLVL
VAFVVVVLSVNKITFMI

>GsSTS45b(Genbank ID: UDP19925)

MSIEQLHPLPSHFRLKDLAAITGRVFELRLNPQEREAATAAHAWFDSHHVYQDLNKH
RFISHAFDSYAGMAFPDADVSHLETCITFFLWAFSFDDLSDEGTFQSNPQAHQAAVDIS
MKVLRNPAAPPPDFPFAAMLHDIWSRFRATASSGACNRFFRAVEGWMRSQVEQIRNR
AIDEIPSVNDFIILRRQTIGGPIVESMIEYSLDLRIPEDVWDHPILQDMSNALIDLMTWPN
DLCSFNKEQADGDFQNLVFCIMIEQDCPLQTAVDILTDMLSQRLVDYEELKAQLPSFGP
EVDAELTRYIKAIEHYTQGTVVWYYSSPRYFRGQAVSGIPEIVVPVYEKSASMQDTTLTP
TAKASKPAPVVKHSPWVKADSSFVHTHVVLQCMIYLLLVLVAFVVVVLSVNKITFMI

>A8411

MSSTKIGKVAPFPPLPGQPWPPARNHPRWKELYRLHDEWLMKHWPFSSEKKRARIPF
MNLAGFSTWCAPASDFDRMVWGGRIAGIFFLADDYIDSGKMLDRIPGFKKAATGEGP
LHPEDRAEICHDIVFRAIKETSHPRTFRQLTQCTHEWWDSNIHEPFRNLDQYLATRRV
NIAMYFANAYFRYCLDINLTDEQVNHPLMREAEGIVSDHVGLTNDLFSYAKEYLTQSD
DTNVLRMLQDFEGLTYEQAKDTVTKKIRQKEQDFIPAGLAVLNDPELGKDPEVHRWI
ANLPYCMGGNNAWSQESGRYNIGDVPGAPPFPSLSFEAEATPEDEVVDDTEESALRD
AVFNVEVIPEPDFTLDIDAIESKGVRTKTQASIPPSLQLMNIPQSRVQHVGVRAIASSQR
TGSDDNSLILKAVASLVRSYEIDVQTVGKVMIYLQNSNIPVHAADLVHLLSQSEIEVNIS
NGDSSALLLDAITHVESNTWDARNVITVAVTKQSVVTMLVGPNAPIVMEPVRGVFTD
NHTQKLFPSSYFTALQKSYESYLKRLRVSLSKEIDLNEATGAASVFDYMLTDHPEKLAE
LFRVSPCLSKDDNALPMHPRDFQKKVVFNGPDNGSIYDGLVCLVDAVPSYEFFEKRIAI
FSESNNGTSTFFCLRAVSDTKYMRDTLKAVGGVPRRT

>ShSTS1

MESVREHIPRLQHFLGEIGYRHTTPPAPTLDFLHAHHHWIHHVLGPMTSWTVAKLNA
LEDSSSTIFERAYPLSDAEMKFVLAKLTAIAIFLDDSLEDEETYDDIGNFAHRVYLGEAQP
TGVLTLYHQGIQELSKMHEGDAVFRGLAVAPWITFIDACMLEKRLLTFDSKLRVSPRDL
GYQRLRNSTDFTSLRAPKATPSEVEVSFPIFLRHKSGIGEAYAAAIFKSSRYQELPLSRFVK
SMPDMIYYIELVNDLMSFYKEQLAGETANLIHLQHQSWKGGQGTGPYGSWTLLDTFS
RLCDETRDAAFRVDELLRLDECEKIANGELRGEEVGLSPMDVTMAAQWREFRDGYVS

WHLECQRYKLDFIKLSTFE
>ShSTS3(JGI ID: 122776)
MSTAKPEIPPMESFPPINVYPREAEIIKYCNNYVAEHFPFNNDAEVKHFNGMEIPAYAC
RVVSFARDHEKMRKVSILIVFYFIFDDWVDKNGMKLDKSTVMALLPPPSEVPIKPPQA
GKMTLSDISAELYGAVRDDMPKADYDRMVNDMMEYLRVQRMAPGYNTLQELLDLR
SGEVGVYVLFRYIIYAMELSVSGKELDDPLVKRAQVLGSEAGVLRNEASSYVKEVNEGS
GAHNVITKLQEWSGCTEKEAMKQVLDAIEKRQEELREMCLKVTEAPHLSEDCKTFVK
TIPYIVAGNTWWHHHSTRYAEGREVTVP
>ShSTS4(JGI ID: 52743)
MSETKVGKVAPFPPLPGAPYPPVRNHPRWKELYRLHDEWMMKYWPFSSEKKRARIPF
MNLAGFSTWCAPAADFDRMVWGARIAGIFFLADDYIDSGKMLDRIPGFKAAATGTGP
LHKEDQAEICHDIVFRAIKATSHPRTFDQLTKCTHEWWDSNIHEPFQNLDQYLAVRRV
NIAMYFANAYFRYTLDINLTDEQVNHPLMREAEGIVSDHVGLTNDFFSYLKEKMTNSD
DTNIIRILMDHEHLSYEEAKTVIEKKIRQKEQDFIGAGMAVLNDPELGKDREIYRWIAN
LQYCMGGNLAWSQESGRYNVGVIDGISFPSLSYAAEPTPEDEVVDDTEESRLRELIFNV
KDIPPPDFTIDDDAIFMTNPHSHLQDNVVPLPRPENVGIIGLEVYFPKRCISIDALEDFD
GVAKGKYTIGLGQQYLAFTDDREDINSFALSAVSSLLEKYNIDPRSIGRLDVGTETLIDKS
KSVKTTLMDLFAASGNHDVEGIDSKNACYGSTAAVLNAVNWIESSSWDGRYAIVFAG
DIAIYAEGPARPVGGAGAVALLIGPDAPLVLEPTHGSYMANTYDFYKPRMESEYPVVD
GPSSVTTYITALDESFKAYQRKVQEGSSRDVPPPYANGANGKASATKSVKLSDFDYSVF
HSPYGKLVQKAYGRLTYHDFVAHPTAPVYRDLPTDILSKDASATLTDKSVEKTFAAASA
SMYKQVVTPSLLISNRCGNMYTGSLYGGLASLLTSIPSYELFDKRISMFAYGSGCASTFFA
IKVRGDTSHIKAKLDLERRLAEMDVRPCEDYVVALKLREETHNAPSYIPNDSDAGLWP
GSYRLEAVDGKYRRSYTVTH
>ShSTS5(JGI ID: 161672)
MELSSLRPFPASFVLPNLANITRQAFNLKLNPHSQSANSAMKSWFKSFHVYDEQKSRE
FLEAGKFDLYAALSFPDADLQHLETCLAFFFWAFSTDDLSDEGDLQSKPEEVQVGVDIS
TSALSSHAPTSLDFPYAAMLQSLFNRIKTTATKGASERFIQAFKDWSSSQVMQSRNRSK
LLLPSVEDFILMRRNTIGAALVEAMIEYSLDLDLPDYVFRDPVVIAMSEATTDIMTWPN
DLCSFNKEQADGDYQNLVCCLMAQYDLGLQDAVDRLVGMISTRVRDYITLKEQLPLF
GAEVDTMLRKYHAALEHYVQGTIVWYYSSPRYFHGEQIIEKESTRIILFSKASSKDCS
>ShSTS7(JGI ID: 167646)
MPHSTVHSHTLISDDSVLLFPDLISYCAYPLRVNPYGRSVADDSERWLLNGAHLSDKKR
KAFLRLRAGDLASMCYPDASAKSLRVVADYMNYLFKLDDWTDEFEAEDVDGMRDC
VLAALRDPLHYETDKAVGKLAKSFFGRFVQHGGPLRTKRFIDTMVLFFRAVRQQALD
RTYDDIPDLESYIALRRDTSGCKPCFALIEFAGGYDLPDAVVEHPSIQILQDATNDLVTW
SNDIFSYNVEQSRGDTHNMVVVLMHEQGLAVQEAIDAVADLCERSIDTFEQTRRSLPS
WGPIVDSNVESYIDGLQNWIIGSLHWSFLTERYFGKDRRDVKRKLFVKLLAKRC
>ShSTS8(JGI ID: 146390)
MPAIIRQFILPDLFALSSAFPDATNPHWKRACTESRDWVNSYRVFSDEERRAFFTQGQS
ELLCSHAYPYAGYEQFRTCCDFINLLFVLDEISDEQTADGAWATGRIFLQVLQDPEWDD
GSKVAQMTRDLRARVVSTGVKPHTFRRLVHMCRDYIASVVEEAGLRERGEVLDIESYIE
LRRNNSAVLTCFALIPYILGIDLPDEVVNDPNFSALNLAAVDMVCWANDIYSYDMEQA
KGLEGNNIMTVLTEMHGLTMQDASDYVGEQYKALMDLFLDNQAALRSFGPSVDAD

VRRYVDAVRHWPRGNLSWSFETPRYFREKREEIENTRVVILRPRPEPKMKSQN
>ShSTS10(JGI ID: 111121)
MGATSTTQIPHSTSLESYILPDLLRLSSPFKASMNPHWATTASESSAWFSSYSIFSDQELTE
FAVCKVELLVAYAYPHANYETFRTCCDFMNLIFAIDEISDMQNGEDAGETGDVFLNAL
RDAEWTDGSALAEMTRDFRARFLRSAGPQSFRRFLKVSEDYIDCVTKEAGYRERSQILD
MESFKHLRRDNSGVPLMLGLLEYTLGIDLPDVVFEDTALSRIYWAAVDMVWWANDV
YSYKVEQAKGLAGNNILSVLMAAKNIDLQDASDYVGDVYAGLMKEYTEAKAELVSKS
FGSKELDAAVKKYVDKMENWPIGNLEWSFASMRYFGSQSGEIKRTRTVMLRPQDVLA
>ShSTS11(JGI ID: 128017)
MAAPESSAWVSSYNLFSDRKRTDFITGSNELLVSHTYPHADYDAFRTCCDFVNLLFVID
EISDDQSGKAARRTGEVYLNAMRDPEWTDGSDLAKMTQQFRARFLRSVGPQSFRRFL
RHSEDYIDCVAKEAEYRERGQVLDMDSFKSLRRENSAIRLCFGLFEFTLGIDLPDSVFED
ETFMKMYWASADMVCWANDVYSYNVEQAKGHSGNNIVTVLMAARDIDMQAASDY
VGEYYAELMEEYMTAKAELASKSFGSRDLDEDVWKYVNAMENWPIGNLEWSFKTNR
YFGTLHDEVKRTRLVVIKPRKVVV
>ShSTS12(JGI ID: 111127)
MQTPPSCESYILPDLLHISQPFKASTNPHWLKAAPESSAWVSSYRIFTDRKRMEFILGSN
ELLASHTYPQANYDTFRTCCDFINLLFVIDEISDDQSGEGARLTGKVFLNAMRDPECTD
GSILAKMTRDFRARLLQSIGAQSFRRFLKILEDYIESVTKEAEYREHGYVLDMEQFKILR
RQNSAVRCAFGMVEHTLGIDLPDVVFEDATFMKVYWAAIDMVWCANVNMFPPPGY
LIQDVYSYSMELAKGDNHSANNIMSVFMAANGVSLQAASDYVGRHYARLMEEYLSA
RAELASKSFGSQELDSDVLKYIDAMENWPIGNIEWSFKTSRYFGPQNDEVKQTLVVKL
KPQEVPA
>ShSTS13(JGI ID: 50042)
MPDLFTAWPWKTTRNPLYLEAKADSDGWLATFGDFFQKYKDCILDCDGKRLRCAGD
VNTLLFVVDDILDYEDDATVKERMRILMDAMENPFKPRPDGEWIVGEMTRQFWERTI
KVSNTLSQERFMEGVRDCMAGMVRESLARKASRQHDSIHGFLKTRRDSAGCNLVFAL
SELEVAVPAEAMKHSQIRELIAIIQDISCIANDTFSYRREQSRGRTEDNIITVVMNNLGTD
IPGAIDWVENHHKELMKEFIEKHDKVPKWGEPTDSKVKNYIESLAGWVWANARWSF
ECRRYMGDEGMEIMEKSRWVRAIPKERI
>ShSTS15(JGI ID: 64702)
MVRSPVSDKFCIPDTLASWPYPRILNPHYAEEKAASAAWTKGFGAFGPKAQDAFDRC
DFKRCRSGCDLMNLFFVIDEHSDTHGEETVRKMKDVVMDAIRNPHKPRPNDEWIGG
EIARQFWERAMCYASEISQRRFIDTFDEYLESVVDQAADRDSARIRDIESYINIRRNTIGA
KPSFVIMEQGMDIPDNVFENEVFQRLRMATIDMLCLGNDIVSYNIEQARGDDSHNIVR
IVMNELDTDVPRAMDWVAQRHTQLEREFFTALSELPTWGEPIDGWVKEYVYGLGNW
VRANDQWSFESQRYFGTKGMEIMKSRWLSVLPKVRPAEVGPQLVDQSLL
>ShSTS16(JGI ID: 73029)
MAVATSVATPVPTPAYSAGRAPAKEKKIYLPDTLAEWPWPRAINPHYAEAKEESQAWA
ASFNAFSPKAQHAFNRCDFNLLASLAYPLATKHGCRSGCDLMNLFFVIDEYSDIAPVE
EVRQQKDIVMDALRNPHKPRPEGEWVGGEVARQFWALTITNASAQSQKHFIETFDEY
LDSVVQQAEDRSESRIRDIQSYIDVRRNTIGAKPSFALLELDMDLPDEVLAHPTIQSLSL
ATIDMLCLGNDIVSYNLEQARGDASHNIITIVMNELNLDVNGAMRWVGDFHKQLEK
QFFEAFNNLPKWGNAELDAQIAVYCDGLGNWVRANDQWSFESERYFGARGLEIMET

KTLAMMPIQRTEALGPQLVDDSI
>ShSTS17(JGI ID: 69906)
MSQTYTIPDTLANWPWKRKINQHYEEVKMESASWARSFHAFSPQAQDAFDRCDFKR
LRTGCDLMNLFFVIDEHSDLSSVADAETQAQIIMNALMNPEKPRPHGEWVGGEVARQ
YWELAIKTATPKSQRRFVAAFDDYMNAVVQQAKDRTHSTIRDIDSYMEVRRKTIGAWP
SFALLELDMDLPEDFMDHPVMHELHVLSISMICLGNVSDIAPLPSDIVSWNLEQSRGD
DTHNIVRIVMNQLDTDINGAMAWVEMYHKELEVKFMDIFTKSQEWNKSMNKDISRY
VEGLGNWVRANDQWSFESKRYFGDRGLEIMSKRTVSMMPKRNDVLNGTQLDIGPVI
VDGSIL
>ShSTS18(JGI ID: 25180)
PRHINPHYQEVKKASAAWAESFGAFNPKAQHAYNACDFKRLRTGCDLMNMFFVFDE
YSDVSSPKDVIQQAAIIMDALRNPYAPRPDDEWVGGEVTRQFWKRAIKTATAGAQRRF
IDAFESYTQSVVQQAKDRHHGFIRDVDSYLEMRRETIGAKPSFVVLQMDMTLPDEVLA
HPVIQQLSALSTDMICLGNRRLIQILWTVQDICSYNVEQARGDDLHNIITIAMNQFDIDI
AGAMDWVVKYHAKLERKFLYLYNNGLPSWGKELDPQVERYVCGL
>HS-HMGS
MSETKVGKVAPFPPLPGAPYPPVRNHPRWKELYRLHDEWMMKYWPFSSEKKRARIPF
MNLAGFSTWCAPAADFDRMVWGARIAGIFFLADDYIDSGKMLDRIPGFKAAATGTGP
LHKEDQAEICHDIVFRAIKATSHPRTFDQLTKCTHEWWDSNIHEPFQNLDQYLAVRRV
NIAMYFANAYFRYTLDINLTDEQVNHPLMREAEGIVSDHVGLTNDFFSYLKEKMTNSD
DTNIIRILMDHEHLSYEEAKTVIEKKIRQKEQDFIGAGMAVLNDPELGKDREIYRWIAN
LQYCMGGNLAWSQESGRYNVGVIDGISFPSLSYAAEPTPEDEVVDDTEESRLRELIFNV
KDIPPPDFTIDDDAIFMTNPHSHLQDNVVPLPRPENVGIIGLEVYFPKRCISIDALEDFD
GVAKGKYTIGLGQQYLAFTDDREDINSFALSAVSSLLEKYNIDPRSIGRLDVGTETLIDKS
KSVKTTLMDLFAASGNHDVEGIDSKNACYGSTAAVLNAVNWIESSSWDGRYAIVFAG
DIAIYAEGPARPVGGAGAVALLIGPDAPLVLEPTHGSYMANTYDFYKPRMESEYPVVD
GPSSVTTYITALDESFKAYQRKVQEGSSRDVPPPYANGANGKASATKSVKLSDFDYSVF
HSPYGKLVQKAYGRLTYHDFVAHPTAPVYRDLPTDILSKDASATLTDKSVEKTFAAASA
SMYKQVVTPSLLISNRCGNMYTGSLYGGLASLLTSIPSYELFDKRISMFAYGSGCASTFFA
IKVRGDTSHIKAKLDLERRLAEMDVRPCEDYVVALKLREETHNAPSYIPNDSDAGLWP
GSYRLEAVDGKYRRSYTVTH
>Hetan2_454193(JGI ID: Hetan2_454193)
MAQKIYIPDTLANWKWPPHLNPHYPEVKRESAAWLASFGAFSPKAQDAFDRCDFNL
LASFAYPLAKKEHLRSGCDLMNLFFVIDEYSDVAEADEVQRQADIVMDALRNPHKPR
PKGEWVGGEVTRQFWELAIKTASPQSQKRFIATFDTYTQSVVQQAADRTHSYIRDIKSY
FEVRRNTIGAKPSFALLELEMDLPDKVIEHPIIQDLSILTIDMLHLGNDIASYNLEQARG
DDSHNIVTIAMNQLKTDVAGAMKWVDNHHKELERKFNESFEKLPKWGEPIDSQVAR
YVDGLGNWVRANDQWSFVSERYFGKKGPEIMKSRWVTLLPKERTEDIGPQVVDDSLL
>BvCS(Genbank ID: KU668561.1)
MSTASSPSLVASEIDSPHHSRTSSPSPTLSPPTSFILPDLVSHCNFPLTYHPAGDEQAAASL
AWMLSFVPHFTPKKVAAMNGLQAGELTAYCYHDCPPERLRVVDDFMNYLFHLDNIS
DGMMAKNTTQLADWVMNAFEWPEKFQPTVNADGEVVEEIAAVKLARDYWSRCIQ
QAKPGVQQRFKSSMNMFFQAVEQQTNDRDGQVVPDLESYIDMRRDTSGCKPVFDLIE
YALGFELPEEVVDHPVIKALNQDANDLVTWSNDVFSYNVEQARGDTHNMICIFMEH

DGCTLQEAIDRVGGLCKQTIDAFVENKARVPSFAHLGPEVDAWTTGYVQGLQDWIVG
SLHWSFMTKRYFQEAGAEVKKTRFVKLLPIEEGRHKHIPPIYASAMVAATA
>Sphst_47084(JGI ID: Sphst_47084)
MPLLSCSQTFRLPPLHETFSVFPDNGLNPNYNACRAQSRAWISKYNVQVCGPKMRAF
MDNCNFELSNAYVYPYAQPAGLRATMDLANILWLYDEYTDMQTGEDAAKAAVTVSK
TLLNPEYDDDTWICHMMRDFYVNHIQKCRPNVAHRFIENFCRYTEVVGTEAKLREKN
EVLDIPGYVALRREISAVRTCFDLVEYCLDLDFPDYVHKDPIFVIGYNAAMDLVFWAND
LFSYNSEQAKGHAAANVVTVIMTSKKMNLQSTVDFIAGFCEALTFQLLDAKRALSLH
EDPTFSRDAVRCLEAFGDWVRGNDAWSFATTRYFGPENKIVKETRIVKLKAPVEESVAL
KE
>Copu2(Genbank ID: XP_007771895)
MSTMDPSEFILPDFFATCPFAFGRTNPHADVVIPEAHAWIVKHVPFVDRKRDEFIQDGF
QDLMPHCYPWAGKETLRTMCYLNNLLFLVDDLTDDMNSDEARGFGESFIRVLNDPAV
HDSSQVMQATREFRSRITGTGVTESRWFGRFLAIFKLYINAVCAEAEDRENKRILDLDTF
TVARRENSAVMVFFAITEYALGIDLPDAVYEDPTFLRVYADSADMVILVNDVFSYNREQ
AKGLDGNNNITVLMQTLDLDLQAAVDHVGEMFSQKMEGCMRGRAMLPSWGVKVD
ADVERFFDALDQWVVGNLEWSSQSPRYLGPEHEEIMRTRRVVLRKVETEIE
>Copu3(Genbank ID: XP_007765978)
MSATPAPTEFILPNLFSVCPLTFGRSNPYYDEVIPEARAWIAKYNPFVDSKRAEFVQGCN
ELLCSRVWPYAGREEFRTCCDFVNLLFVLDELSDDMGGADARSTCDSFIRVLNDPDAP
DTSLIAQMTREFRARVAERAKPGCLRRFIALCGTYVEAVCVEAELREQGRVLDLRSFILL
RRENSAVRCCLALAEYALGLELPDAVFNDPAFQSVYFCAADMVCWSNDVYSYNMEQ
AKGHTGNNVVTVLMQEHGIDLQAAADRVGEVFGQLMEHYTSGSRSLPTWGGKVDA
DAARFLEAAGQWVVGNLEWSFETPRYFGPDHDEVRDTHRVLLK
>Copu5
MHLPEPFHFLLPDFSSHCSYPLRLNKHCVAAAAASEDWLIRLAQLRSPRNGRKLKKFM
GLKAGYLTALCYPDCPRTELRVVSDYMNFLFTLDDWSDEFAEAGVRGLEQCVMGMLY
DPTVKTDKAAGRLARSFWLRMIRTAGPRVQHRFIVAFEDFFRAVEQQSRDRAKGVMP
DLESYIALRRDTSGCRPVFVLAEYAAGIELPDEVFEHPIIQSMTEATNDLVTWSNDVFSY
NKEQALGDTHNMITLLMAQHGLSLQGAVDFVGQLCAASITRFESGRTTLPSWGPDVD
CDVQKYVMGLQDWIAGSLHWSFETERYFGKRGKEVRQAGVVKLSPMKAPKKV
>Copu9
MSPTATFTTTSSEENAPTKFILPDLVSDCTYPLLLNDNCEPVARASEQWLIAGARLQEPR
RTKFMGLAGELTACYPHADASHLRVCVDFMNWLFNMDDWLDDFDVDDTWGMRH
CCLGAFRDPVGFETDKLGGLMSKSFFSRFRQDGGPGCTERFIHTMDLFIAVAQQAGDR
ANGITPDLESYITVRRDTSGCKPCFALIEYAAGIDLPDHVIYHPTLAAMEEATNDLVTW
SNDIFSYNKEQVTDDTHNMIPVLMRERGLDLQGAVDFVGRLCKGTIERFETERARLPS
WGPELDAQVQTYIEGLQNWIVGSLHWSFDSHRYFGKDGHAVKKHRIVKLLPKRVPQ
QA
>PCSTS01
MQTQQTSSVVSSSPRKIIIPDLVSHCTFPLRNNRHRKQATVECKRWLFKGGNLSQKKRD
AFHGLKAGLLTSMCYPNAAFPQLRVCCDFMNWLFHMDNISDDMTDRGTANTGVDV
MNALWLLDDYTPTTRVGKMARDFWRRAAVTAAPGAQQRFRETMDFFFQSVTQQAL
DREAGVVPDLESYIALRRDTSGCKPCWALIEYAYNLHLPDEVMEHPTILALGEAANDL

VTWSNDIFSYNVEQSKGDTHNMIVVVMQQDGLDLQSAVDFVGDLCQQSIDRFVYER
DNLPSWGPEIDRQVDIYVDGLRDWIVGSLHWSFESERYFAKSGLEVKKTRTVNLLPLRT
AVAEEPTQI
>PCSTS02
MQSFVLPDLFAVCPLVGSTNPHYAKAARESSAWIDGYRLFTDRKRAFFIQGSNELLVSH
TYPYADYEQFRTCCDFVNLLFVVDEVSDDQDGKGARSTGEVFLNVMCDPAWDDGSA
LAQMTREFRARFMQYAGPNCARRFLVHCKDYVEAVAREAELRETGEILDVEAFKHLR
RENSAIRLCFGLFEFALGVDLPDAVFEDETFTKLYWAAADMVCWANDVYSYKMEQAR
GIDGNNIVTVLMHERGVDLQTAADLVGEHFARLMDTFLQTKRALPSWGLTLDTAVAA
YVAAMEHWVIGNLEWSFETQRYFGPTHAEIKRTRVVELRPTEDAFDGDD
>PCSTS03
MSTYPALRSYRLPDLHPLCPFKARFNPHHEEAAAASKAWVLSFNALKGKKLEFFKEG
GSELLCAWAYPYASLEGLRTACDFVNLLFTIDEISDEQNGNDALATGMSVWNTMKDD
NYDDGTVLCRMTKDFKKRFFPYAGPATTRRFLKHTEDYVLGFAREAELREKNVVLSLA
AYDPLRRENSAVRYCFGLFGYLLGMDLPDEIFEHPLYMEMHLAAVDMVCWANDVYSY
DMEQSMGHLTNNILTVLQREKNIDLQAASDYVGVHFKELADKFEANKALLPSFGKEL
DDVVAHHIMAMEAWVAGNLEWSFGTRRYFGKNHMKVRETLVVELSPPRVLDD
>PCSTS04
MPAIAPPSAPATYRLPDLHAVCGFKARFNVHYEEATAGSKAWYFSYKTLTGKRLEFFKE
GGSELLCAWAYPYAGLGQLRAACDFVNLLFTVDEISDAQNGVDALATGMSFYNTLKD
DNYDDGTVLCRMTKDFKKRFFPYAGAATARRFLEHTKNYVLGFAREAELREKNVVLD
LLASYEPARRENSAVRYAFGLFGYILGMDLPDEIFEHPIYMEMHLAAVDMVCWSNDVY
SYDMEQSLGLAANNILTVLQREKNVDLQTAADLVGSHFKVLVDKFEAAKARLPSFGK
ELDEIVAHHILAMESWVAGNLEWSFGTRRYFGKSHLKVRETLVVELSPPKYLGDL
>PCSTS06
MAPVIGTIEMPPASDGFGNPAKISPTHERSEEESLPYLCRYVITEFLRRAGIQMPTFNAAS
FGDEVDRLTFAEVAKWDIGNANPRRLHHHVVSAINIAKTAYAHTPVPTQVHIAMWTA
LCIFVDDFEIETAAVEQFAERFHAGGPQLHPLLDVFAGTLRAMPRFFHAHGAAGIVAN
TVQYVTSTLFDKVTEEKGLEVHASARDYPLYKRARNGIGEGYGFCIFDKENFPDVSTHI
QVIPEAITYLIYVNDLLSFYKEELDGETKNFIHDRARVTGKDIEAALMDSMEDVIDAVN
RGRQILQGEKERKAWDSFLVGYVAFHFISPRYKLERLLSGMN
>PCSTS08
MLVDLAGQLQARLRALVGVEYFKDFVAVNDERAGTEETCRNALRCFVGRVKIPARVM
QPDPTGSVTQSLRATVKAWGCIDTTSAACQKRVAAAVAITTLVFRHTRLDTQAYIAGY
AFLAISLDDEAIGADALAAFAPRMLSGEPQGHWVLDRFIEHVQSAPTFFGAFSVAAIA
ACTVQFVNSTLDRTTSNRVCLTAGSLPYVLYKRAINGVGDSFALFAWEKDRFPDDSLFL
QVLPDLCRFMEYTNDILSFYKEELAGEKDNFVHDIMTVSELPAPGVLEGLVDAAAETV
QRARLVLQDSEERKVFESMLEGYVAFHYTSPRYRLGDLSGASPADSD
>PCSTS11
MCATLTDFLWQAEAAKHAVDSLLLRSAPLPEWKTMTFDADGTLERRIREVVADWEPA
VTSLQGFERSILISAALGMSFNSHSPMEVRLQVALYSLVIFLTDDCEIPAAALDEFMHRFY
SGRPQLHPVLDHMVEILHGMDEFFPPLAVKGIVQGTVDYMNVNAFEPHAEKLPLHPA
ALSYVTARRMKHGLAEPFLMFLFDKFNYPDVSTWVQAVPDMMIYTNWANDIYSFHK
EILADDVHTYILERAEVTGKDLTETLSEIVDEAVVACDNVRKILKGTKALEAWESFVAG

YAAFHRYTPRYRLKELYGDEERDRM
>Denbi1_659367(JGI ID:Denbi1_659367)
MTVPIPTSTEQSSAPTRFFIPDTLANWPWPRALNPAYEQCKADSAAWCEKYKAFSPKA
QKAFNLCDFNLLASLAYAHLPEDVNRVGCDLMNLFFVVDEHSDAMDKNSVHVWVEI
IMDALRNPTKPRPDDEPIVGEISRTF
WENAIKCLGPTSQKRFIETFETYLYAVIVQADDRDHHVFRDVDSYMVVRRDTIGAKPSF
ALLEHNMDLPDDVFNHPLLEDLRTWCIDMLILGNDLCSYNVEQSRGDDGHNIVKLV
MLQENIDLHGAMQYISDMHDDLADK
FLRNYKNMPSWGQPIDEWVTRYIEGLGNWVRANDAWSFESWRYFKYDGLRIQKERW
VELLPPANKEDLTSSSE
>SiTPS(JGI ID: 77541)
MPSVSPATIRLPDILGAMDRFELRTHPDEREVTRASNEWFNSYNMMPPALFEKFVKCD
FGLMTGMSYPDTDATRLRITCDYMSILFAYDDLMDLPSSDLMHDKIASDKAAKIMMG
VLTHPHKFRPYAGLPVATAFHDFWTRFCATSTPKMQKRFTDTTYEYVMAVKNQCGNR
QSSRCPTIEEYVALRRDTSAIKVTYACIEYCLNIDVPDEAFYHPSVAALQEAGNDILSWA
NDVYSFDNEQSSGDCHNLVAIVAINKNITVQAAMEYVMGMIDSAIERFFEECANVPSF
GPEVDPLVQAYIKGVELYLSGSVFWHLESSERYFGARVQHVKDTLMVELRPLDEGAKP
AFDLMYKLPSNLTPEVLSAAAVSAAPAAPAPVASPAPQPEILSPTPISPINVNFPLGNVA
CPPPSYETQRVLAKMVATVEEKQRLAYSQPAEQYYSPAPQYYPSQPVEKFQQTNVLETA
FKGSNSELTNILVIASVLMAGSPMALVPFVPLLALLLLPNETPVAPVA
>SbTps1
MVNSSSSPSPLLVPLLVPLPSPSTVRSSTKPLPEGFILPDLVSHCKFPLSYNPHGDVVAAQ
ADIWLDKGCPELTPKMRKALYGLHAGELTAYCYTSCDAHRLRVISDFMNYLFHLDNIS
DGMMTRDADVLSDIVMNALVFPHEYKPCKGQPAEEISAGKLARDYVLRCIADAGPDV
QARFKENLQLFFEAVHIQATHREDGEIPDLETYIDVRRDESGCKPVFDLIEYGLGINLPD
FVIEHPIIKALNQGSNDLVTVSNDIFSYNVEQARGDTHNMIILVQRYGMELQDAMDYV
GEMMCRVTMENFVANKARLPSFGCPELDRDVAGYVQGLQDVIVGALHWSFMSQRYF
GTEGAEGAEVKKHRYVKLLPKKCTTTPAAGTETRSPECLSQTADDIETTYLPTRLIYQA
GRSMRNIASWLLQFFSRRHEVCS
>Denbi1_816208(JGI ID: Denbi1_816208)
This sequence is not available.
